# Immune mediator expression signatures are associated with improved outcome in ovarian carcinoma

**DOI:** 10.1080/2162402X.2019.1593811

**Published:** 2019-03-28

**Authors:** Mano Nakamura, Heather J. Bax, Daniele Scotto, Elmira Amiri Souri, Sam Sollie, Robert J. Harris, Niklas Hammar, Goran Walldius, Anna Winship, Sharmistha Ghosh, Ana Montes, James F. Spicer, Mieke Van Hemelrijck, Debra H. Josephs, Katie E. Lacy, Sophia Tsoka, Sophia N. Karagiannis

**Affiliations:** a St. John’s Institute of Dermatology, School of Basic and Medical Biosciences, King’s College London, London, UK; b School of Cancer and Pharmaceutical Sciences, King’s College London, London, UK; c Department of Informatics, Faculty of Natural and Mathematical Sciences, King’s College London, London, UK; d King’s College London, School of Cancer and Pharmaceutical Sciences, Translational Oncology & Urology Research (TOUR), London, UK; e Unit of Epidemiology, Institute of Environmental Medicine, Karolinska Institutet, Stockholm, Sweden; f Unit of Cardiovascular Epidemiology, Institute of Environmental Medicine, Karolinska Institutet, Stockholm, Sweden; g Departments of Medical Oncology and Clinical Oncology, Guy’s and St Thomas’ NHS Foundation Trust, London, UK

**Keywords:** Immune activation, ovarian cancer, immune mediators, inflammation, Th1/Th2/Th17, M1/M2, inflammation, biomarkers

## Abstract

Immune and inflammatory cascades may play multiple roles in ovarian cancer. We aimed to identify relationships between expression of immune and inflammatory mediators and patient outcomes. We interrogated differential gene expression of 44 markers and marker combinations (n = 1,978) in 1,656 ovarian carcinoma patient tumors, alongside matched 5-year overall survival (OS) data in silico. Using machine learning methods, we investigated whether genomic expression of these 44 mediators can discriminate between malignant and non-malignant tissues in 839 ovarian cancer and 115 non-malignant ovary samples. We furthermore assessed inflammation markers in 289 ovarian cancer patients’ sera in the Swedish Apolipoprotein MOrtality-related RISk (AMORIS) cohort. Expression of the 44 mediators could discriminate between malignant and non-malignant tissues with at least 96% accuracy. Higher expression of classical Th1, Th2, Th17, anti-parasitic/infection and M1 macrophage mediator signatures were associated with better OS. Contrastingly, inflammatory and angiogenic mediators, CXCL-12, C-reactive protein (CRP) and platelet-derived growth factor subunit A (PDGFA) were negatively associated with OS. Of the serum inflammatory markers in the AMORIS cohort, women with ovarian cancer who had elevated levels of haptoglobin (≥1.4 g/L) had a higher risk of dying from ovarian cancer compared to those with haptoglobin levels <1.4 g/L (HR = 2.09, 95% CI:1.38–3.16). Our findings indicate that elevated “classical” immune mediators, associated with response to pathogen antigen challenge, may confer immunological advantage in ovarian cancer, while inflammatory markers appear to have negative prognostic value. These highlight associations between immune protection, inflammation and clinical outcomes, and offer opportunities for patient stratification based on secretome markers.

## Introduction

Ovarian cancer is a lethal gynecological malignancy with 5-year survival rates of <48% and few improvements in clinical outcomes in the last decade.^1,2^ Complex interactions between immune and cancer cells in the tumor microenvironment involve multifaceted contributions of associated secretomes,^3^ including critical roles in cancer progression and survival.^4,5^ However, comprehensive evaluations of immune mediator signatures in relation to disease progression are still required to help inform prognostic or predictive algorithms for disease management.^6^

The immune system is capable of locating, recognizing and ultimately eliminating tumor cells,^7^ and leukocytes play a major role in these processes. For example, T helper lymphocytes stimulate antigen-specific effector cells, enhance cytotoxic immunity and recruit inflammatory cells to tumor sites.^8^ Macrophages can destroy cancerous cells by innate mechanisms that involve secreted mediators such as interferon gamma (IFNγ), granulocyte-macrophage colony-stimulating factor (GM-CSF), and tumor necrosis factor alpha (TNFα).^9^ Macrophages can also be activated by antibodies to trigger antibody-dependent cellular cytotoxicity (ADCC) or phagocytosis (ADCP).^10–12^ On the other hand, tumors evolve strategies to evade immunological control, including reduced antigen presentation, upregulation of anti-apoptotic molecules, modified cancer antigens arising from genomic instability and co-opting immune cells to promote tumor proliferation and spread. Ovarian cancer cells and tumor-associated fibroblasts may also secrete mediators (e.g. IL-10, VEGF, TGFβ) which may moderate or modulate immune cell activation, promote regulatory T lymphocytes (Tregs) and alternatively activated M2 macrophages, to promote inflammation and wound healing effects rather than activate classical immunity.^13^

Such juxtaposing contributions suggest that there is a critical balance between anti-tumor and pro-tumor environments, which may often be tilted in favor of tumor growth and escape from immunological surveillance.^14,15^ Previous studies have identified specific immune mediators and pathways in patient sera, ovarian carcinoma ascites and tumor lesions that may hold predictive or prognostic value.^14,16–20^ We hypothesized that a broader evaluation of secreted immune, inflammatory and angiogenic mediator signatures, reflecting the tumor-associated immunological environment, may associate with differential clinical prognoses. In this study, we selected 44 immune, inflammatory and angiogenic mediators known to be involved in immune response to pathogens. We analyzed associations with ovarian cancer by machine learning methods in 954 samples that include 839 ovarian cancer tissues and 115 non-malignant ovary samples. Furthermore, we evaluated the prognostic impact of differential gene expression of 1,978 immune and inflammatory mediator signatures from genomic expression data of 1,656 ovarian carcinoma patient tumors in silico. We also examined serum inflammation mediators in 289 subjects diagnosed with ovarian cancer from the Swedish AMORIS cohort.^21^ With these, we explored any associations between different immune, inflammation and angiogenesis mediator expression profiles, with overall survival (OS).

## Results

### Identification of mediators with known or putative roles in protective immunity and cancer-associated immune responses

**Figure 1.**
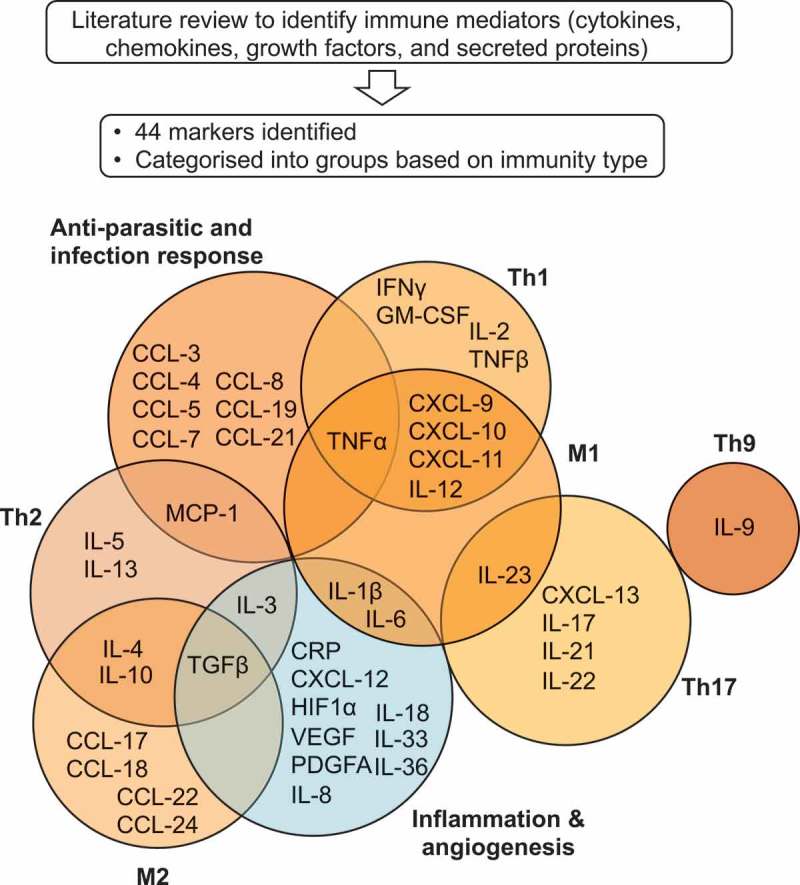
Schematic representation of immune and inflammatory mediators selected and categorized by immune response type. A literature search was conducted to identify secreted immune mediators associated with different categories of immune activation with known possible roles in cancer immunity. This identified 44 immune mediator markers (cytokines, chemokines, soluble inflammation and angiogenic factors), categorized into the following groups: Th1, Th2, Th9, Th17, M1 macrophage, M2 macrophage, anti-parasitic/infection response (immune activation genes, in orange), and, angiogenesis and inflammation (in blue) for evaluation of associations with patient survival in ovarian carcinoma.

We aimed to determine whether relative elevated gene expression of immune-associated secreted mediators in patient tumors was associated with ovarian cancer patient survival. A literature search was conducted to select primary pre-clinical and clinical studies in rodent models (mouse, rat), human cell lines, organoid/3D models, and/or ex vivo evaluations with primary human cells. Mediators (cytokines, chemokines, growth factors, and secreted proteins) were selected and categorized based on the following criteria: (a) reported association with known immunological protection from pathogens, (b) evidence of association with and/or contribution to specific immunity type (e.g. Th1/2/9/17, inflammation/angiogenesis); (c) shown to play specific roles (pro-tumoral/anti-tumoral) in rodent and human xenograft models of cancer, in ovarian carcinomas and/or other tumor types in different contexts (e.g. pre-clinical/clinical experimental interventions such as prophylactic or curative treatments, depletion or blockade with antibodies, siRNA/shRNA, knock-out studies; studies of clinical data and human samples reporting associations with patient outcomes). This search identified 44 immune mediators (cytokines, chemokines, mediators of inflammation and angiogenesis) associated with distinct leukocyte subsets and immunological responses and with known possible roles in cancer immunity (Supplementary Table 1). Selected mediators were categorized into specific immune signatures, namely Th lymphocyte subsets, anti-parasitic/infection responses, macrophage polarization states, and inflammation and angiogenesis (Figure 1).

### A signature of combined immune and inflammatory mediator expression can predict ovarian cancer from non-malignant ovarian tissues

**Figure 2.**
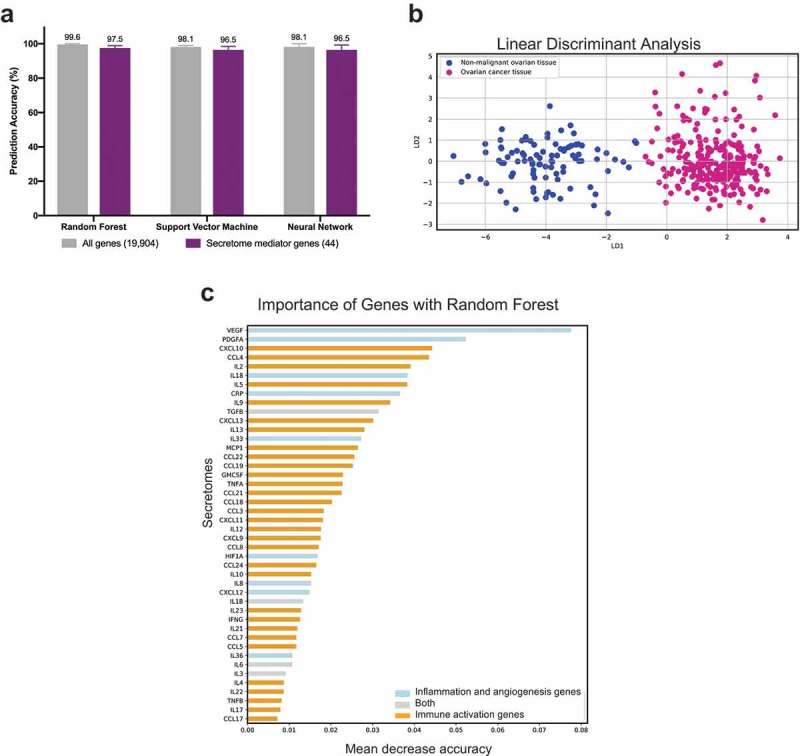
Immune and inflammatory mediator signature can discriminate ovarian cancer from non-malignant ovarian tissues. (a) Comparison of machine learning prediction accuracy for classifying healthy and ovarian tissue samples using three methods (Random Forest, Support Vector Machine and Neural Network.). Comparisons were performed across all 19,904 genes (‘All genes’) and using the 44 selected genes (‘Secretome mediator genes’) as features. SMOTE was applied to account for the imbalance in the sample size of the training dataset of a total of 954 samples (115 samples for non-malignant ovarian tissue and 839 samples for ovarian cancer tissue), and a 5 times 10 fold cross-validation was performed. The average of 5 runs is shown ± the range of accuracies derived across 5 runs. (b) Linear Discriminant Analysis (LDA) plot to illustrate discrimination of non-malignant and ovarian tissue samples with the 44 mediator genes. (c) Ranking of 44 mediator genes using Random Forest feature importance with higher rank, suggests better discrimination across the two disease classes.

Machine learning models were used to investigate whether the 44 mediators selected by literature search were able to distinguish between non-malignant tissues and ovarian cancer tissues. Three methods, Support Vector Machine, Random Forest and Neural Network, were used. The set of 44 selected mediator genes (‘Secretome mediator genes’ in Figure 2(a)) were able to discriminate between two classes (non-malignant vs cancerous) with similar evaluation metrics when all 19,904 genes (‘All genes’ in Figure 2(a)) in the dataset were used as prediction features (other metrics such as recall, sensitivity, specificity, Matthew correlation coefficient, and F1-score are reported in Supplementary Table 2). Although using ‘All genes’ gave almost 100% accuracy in average for predicting ovarian cancer, our ‘44 mediator genes’ also had more than 96% accuracy on average. The feature importance in the Random Forest analysis identified that VEGF and PDGFA were the most discriminant features among our 44 selected genes in discriminating ovarian samples from controls (Figure 2(c)). Additionally, Linear Discriminant Analysis (LDA) using our 44 mediator genes, showed clear segregation of ovarian cancer and non-malignant tissues (Figure 2(b)).

Together, these findings highlight that the 44 gene signatures selected for this study could distinguish non-malignant ovarian tissues and ovarian cancer well.

### High intratumoral expression of “classical” Th mediators is associated with better 5-year overall survival

Using publicly available genomic datasets, we assigned tumor specimens from 1,656 ovarian carcinoma patients into two cohorts based upon the relative mRNA expression levels of an immune mediator or mediator signature of interest. The top 25% of patients whose tumors expressed the highest levels of a given mediator or combination of mediators were classified as high expressing, while all other subjects were allocated to the low expression group. The 5-year OS in the high and low expression cohorts was compared. A follow-up of 5 years was selected based on the low (<45%) 5-year survival expectancy for ovarian carcinoma patients at diagnosis. Survival analyses were carried out for the 44 markers identified by literature search, and for combinations of up to four markers from each immune category. A total of 1,978 tests, of the 44 markers or in combinations were examined in this study (Supplementary Figure 1; all HR and *P*-values illustrated in Supplementary Figures 2, 3 and 4).

**Figure 3.**
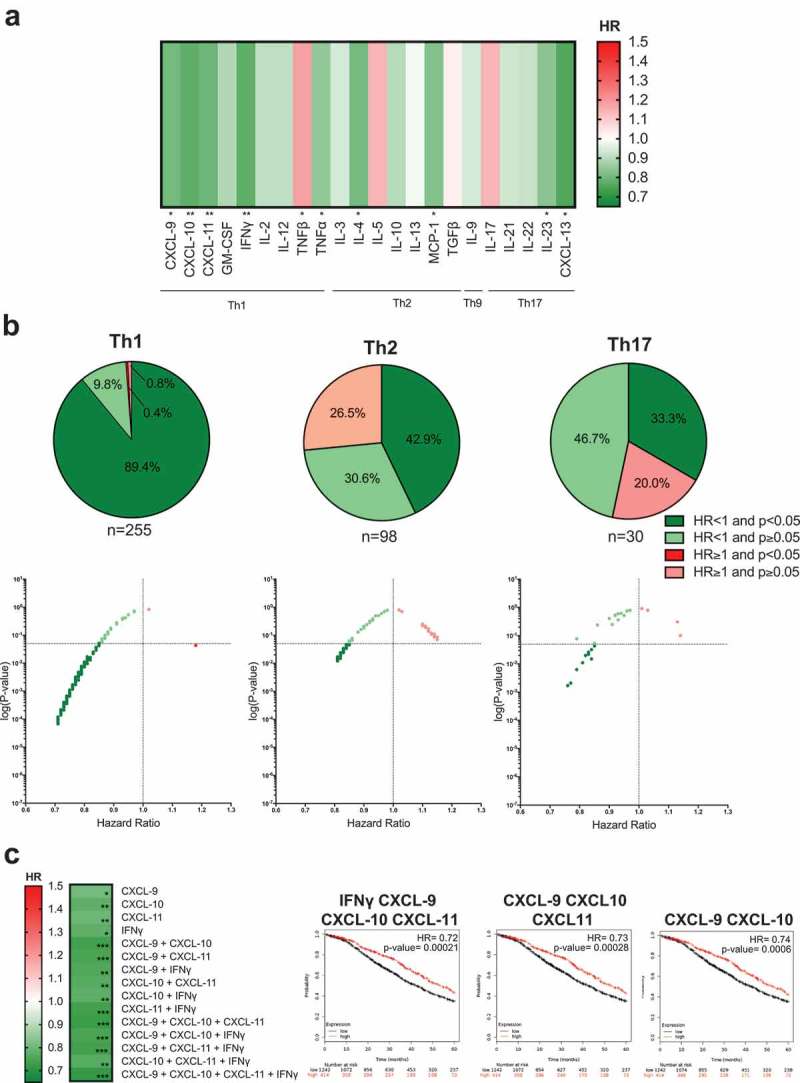
Elevated expression of mediators associated with Th1, Th2 or Th17 lymphocyte subsets is associated with improved 5-year overall survival in ovarian cancer. (a) Heatmap illustrating the association of elevated intratumoral expression of 22 individual cytokines in Th1, Th2, Th9 and Th17 categories, with 5-year overall survival of ovarian cancer patients. HR < 1, indicates better survival of patients (depicted in dark green). HR > 1 indicates disadvantage to survival of patients (red). HR of 1 indicates no prognostic value. (b) All combinations tested within Th1: n = 255, Th2: n = 98, and Th17: n = 30, are shown as pie chart and dot plot graphs. (c) Heatmap illustrating the patient survival when up to four mediators that are independently associated with better prognosis (CXCL-9, CXCL-10, CXCL-11, and IFNγ) are combined. The three combinations associated with the best prognosis are highlighted on Kaplan-Meier survival curves. (*P*-values: * = *P* < 0.05, ** = *P* < 0.01, *** = *P* < 0.001, **** = *P*< 0.0001).

We evaluated whether high intratumoral expression of immune mediators of the “classical” Th1, Th2, Th9 and Th17 responses may have any associations with clinical outcomes. Examination of individual mediators indicated that 9 out of the 22 mediators: CXCL-9, CXCL-10, CXCL-11, IFNγ, TNFα, IL-4, MCP-1, IL-23, CXCL-13, were significantly associated with better patient survival compared with the lower genomic expression group. Only one marker in this cohort, TNFβ, was significantly associated with worse prognosis (Hazard Ratio (HR) = 1.18, *P*= 0.043) (Figure 3(a), Supplementary Figure 2A). The cytokines with the most marked associations with better survival were CXCL-10 and IFNγ, associated with 22% lower risk of death (CXCL-10: HR = 0.78, *P*= 0.0037; IFNγ: HR = 0.78, *P*= 0.0036). Furthermore, subjects with higher intratumoral expression of Th2 cytokines, IL-4 and MCP-1, showed superior survival with 19% and 17% reduction in risk of death, respectively (IL-4: HR = 0.78, *P*= 0.012; MCP-1: HR = 0.83, *P*= 0.029). Associations of elevated expression of Th17 cytokines, IL-23 and CXCL-13, with better 5-year OS were also observed, with 16% and 25% reduction in risk of death, respectively (IL-23: HR = 0.84, *P*= 0.015; CXCL-13: HR = 0.76, *P*= 0.0017).

Overall, 89.4%, 42.9% and 33.3% of all combinations of up to four mediators tested in the Th1, Th2 and Th17 categories, respectively, were significantly associated with better patient survival (Figure 3(b), Supplementary Figure 2B-D). In these categories, TNFβ was the only marker out of a total of 255 analyses conducted with single and combined mediators that was significantly associated with worse patient prognosis.

The greatest association with improved survival was observed with high expression of Th1 cytokines (IFNγ (HR = 0.78, *P*= 0.0036), CXCL-9 (HR = 0.81, *P*= 0.012), CXCL-10 (HR = 0.78, *P*= 0.0037) and CXCL-11 (HR = 0.8, *P*= 0.0078)), and also when these mediators were combined (Figure 3(c)): a combination of two mediators: CXCL-9 and CXCL-10 was associated with an HR of 0.74 (*P*= 0.0006); the combination of CXCL-9, CXCL-10 and CXCL-11 was associated with an HR of 0.73 (*P*= 0.00028); and the highest improvement in patient survival was measured with higher expression of all four mediators (IFNγ, CXCL-9, CXCL-10, and CXCL-11) (HR = 0.72, *P*= 0.00021) (Figure 3(c)). These may point to synergistic functions of these mediators that may confer survival benefits.

Together, these findings identify immune mediators of “classical” (Th1, Th2, and Th17) responses, known to be involved in protective immunity to bacteria and viruses, and also those involved in pathogenic conditions such as allergies and autoimmunity, that were associated with better prognosis in the context of ovarian cancer.

### High intratumoral expression of anti-parasitic/infection immune mediators may confer improved patient survival

**Figure 4.**
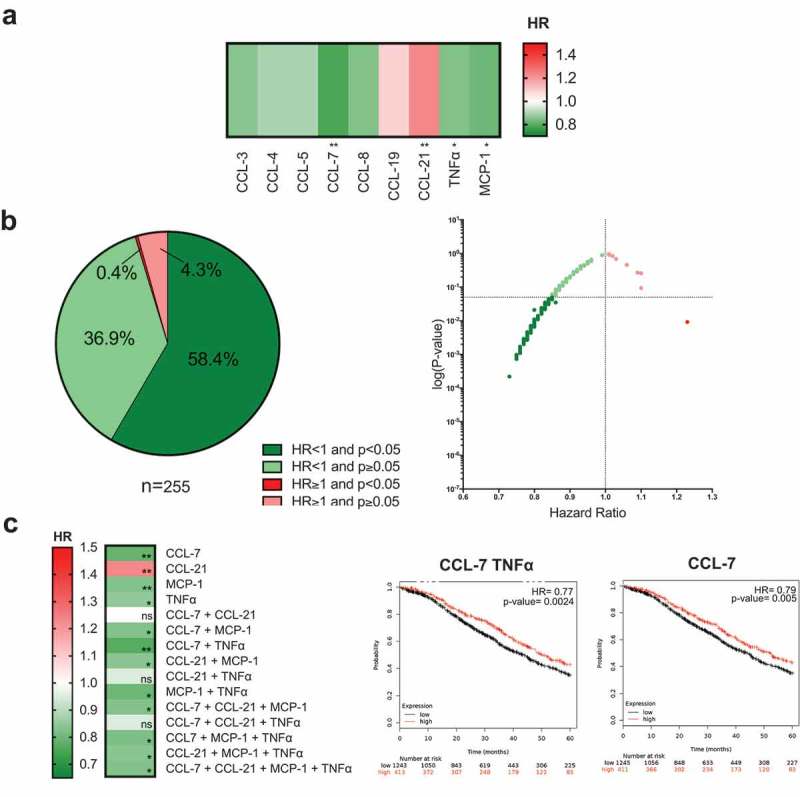
High expression of anti-parasitic/infection response mediators is associated with better patient survival. (a) Heatmap illustrating the association of elevated intratumoral expression of 9 individual cytokines in the anti-parasitic clearance category, with 5-year overall survival in ovarian cancer patients. HR < 1, indicates better survival of patients and (dark green). HR> 1 indicates disadvantage to survival of patients (depicted in red). HR = 1 indicates no prognostic value. (b) All tested combinations of mediators involved in anti-parasitic/infection response (n = 255) shown as pie chart and dot plot. (c) Heatmap illustrating the patient survival when up to four mediators that are independently associated with better prognosis (CCL-7, CCL-21, TNFα, and MCP-1) are combined. The three combinations associated with the best prognosis are depicted in Kaplan-Meier survival curves. (*P*-values: * = *P* < 0.05, ** = *P*< 0.01, *** = *P*< 0.001, **** = *P*< 0.0001).

TNFα and MCP-1 are pro-inflammatory mediators, part of the Th1 and Th2 secretomes, respectively, but are also involved in anti-parasitic/infection immune surveillance.^22–24^ We sought to investigate whether high expression of these and other mediators involved in these immune responses (n = 9), could have prognostic value. In addition to TNFα and MCP-1 (TNFα: HR = 0.85, *P*= 0.046; MCP-1: HR = 0.83, *P*= 0.029), high expression of the macrophage chemoattractant, CCL-7, was also associated with improved patient 5-year OS (HR = 0.79, *P*= 0.005). One marker of the nine evaluated, CCL-21, was significantly associated with worse prognosis (HR = 1.23, *P*= 0.0092) (Figure 4(a), Supplementary Figure 3A). Of the 255 combinations of up to four mediators tested, 58.4% were significantly associated with better OS, 36.9% were associated with an HR<1, and 0.4% were significantly associated with worse prognosis (Figure 4(b)).

When the three mediators most significantly associated with better survival, CCL-7, TNFα, and MCP-1, were combined, the best patient prognosis was associated with a combination of high expression of CCL-7 and TNFα (HR = 0.77, *P*= 0.0024, reduction in risk of death of 23%) (Figure 4(c)). Furthermore, combination of any of these three mediators with high CCL-21 expression, which was independently associated with worse patient prognosis in our evaluations, was often associated with an improved patient survival (Figure 4(c)).

These findings provide support that immune signatures known to be associated with responses to parasitic, viral or bacterial infections may contribute to better patient survival in ovarian cancer patients.

### Elevated M1-type macrophage expression signatures are associated with better patient outcomes

**Figure 5.**
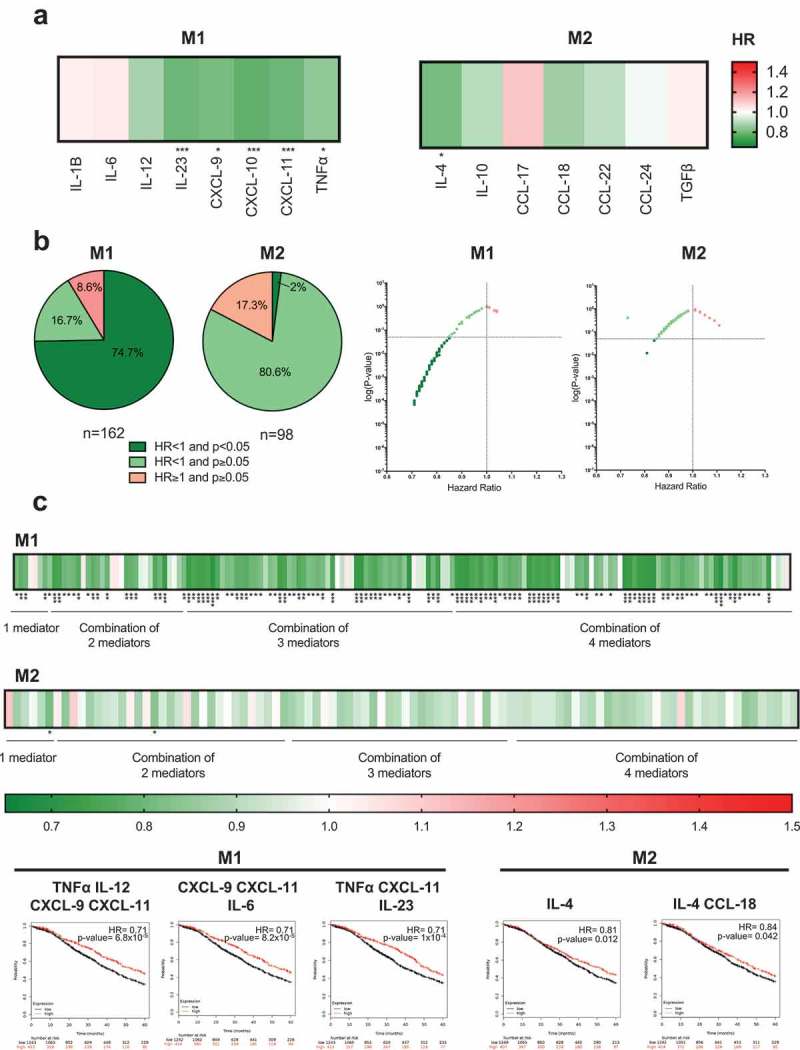
High expression of intratumoral M1 markers are more likely to be associated with improved survival than M2 markers. (a) Heatmaps illustrating the association of elevated intratumoral expression of M1 and M2- type macrophage-related mediators, with 5-year overall survival in ovarian cancer patients. HR < 1, indicates better survival of patients and (depicted in dark green). HR > 1 indicate disadvantage to survival of patients (depicted in red). HR = 1 indicate no prognostic value. (b) All tested combinations within M1 (n = 162) and M2 (n = 98) shown as pie chart, dot plot and heatmaps. (c) The three combinations from each category associated with the best prognosis are depicted in Kaplan-Meier survival curves. (*P*-values: * = *P*< 0.05, ** = *P*< 0.01, *** = *P*< 0.001, **** = *P*< 0.0001).

Macrophages comprise a substantial immune infiltrating population in ovarian carcinoma lesions; however, their contributions to anti-tumor responses have been long debated. We analyzed immune signatures linked to classically activated M1- and alternatively activated M2-macrophages, to assess whether differential macrophage polarization states might affect cancer progression and patient survival. Elevated tumor expression of M1-associated mediators was more likely to be associated with improved prognosis than M2-related signatures (Figure 5(a)). Overall, 74.7% of the eight M1 mediators and their combinations, were associated with significantly improved 5-year survival, while only 2% of the seven M2 mediators and their combinations had any significant associations with improved survival (Figure 5(b,c)). The remaining 80.6% of M2 mediator combinations showed HR lower than 1. For both M1 and M2 signatures, some combinations showed an HR greater than 1 (8.6% and 17.3%, respectively) (Figure 5(b), Supplementary Figure 3B and 3C). Combining beneficial M1 mediators was associated with a reduction of risk of death by as much as 29% (e.g. TNFα, IL-12, CXCL-9, CXCL-11: HR = 0.71, *P*= 0.000068). Combining M2 cytokines did not significantly alter the risk of death, and the best patient outcome was associated with high expression of IL-4 alone (HR = 0.84, *P*= 0.042, reduction of risk = 16%) (Figure 5(c)). Overall, none of the M1 or M2 mediator combinations were significantly associated with worse survival in ovarian carcinoma.

These analyses suggest that high levels of M1-associated signatures are more likely to confer a survival benefit in ovarian cancer, while a markedly lower proportion of M2 mediator combinations are positively associated with better patient outcome.

### Inflammatory and angiogenic mediator expression in patient tumors shows negative or no association with patient survival

**Figure 6.**
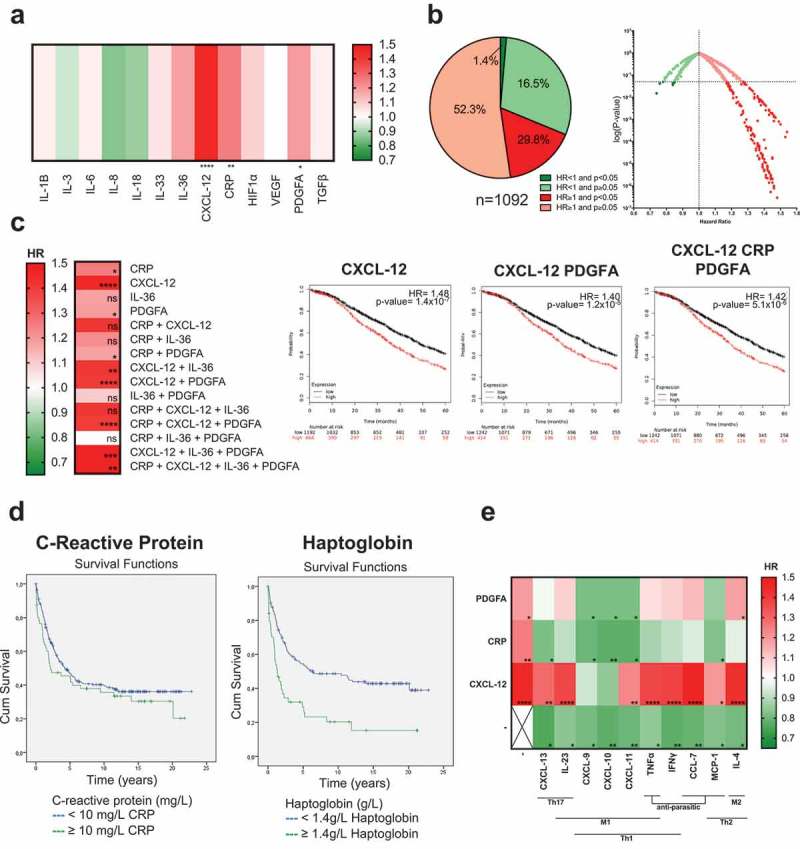
Elevated expression of inflammation and angiogenic markers is associated with no effect or worse survival. (a) A heatmap illustrating the association of elevated intratumoral expression of 13 individual cytokines of inflammation and angiogenesis, with 5-year overall survival in ovarian cancer patients. HR < 1, indicates better survival of patients (depicted in dark green). HR > 1 indicates disadvantage to survival of patients (depicted in red). HR = 1 indicates no prognostic value. (b) All tested combinations within inflammation and angiogenesis category (n = 1,092) shown as pie chart and dot plot. (c) Heatmap illustrating the patient survival when up to four mediators that are independently associated with better prognosis (CRP, CXCL-12, IL-36, and PDGFA) were combined, and three example Kaplan-Meier survival curves of signatures associated with the worst prognosis. *P*-values: * = *P*< 0.05, ** = *P*< 0.01, *** = *P*< 0.001, **** = *P*< 0.0001). (d) Kaplan-Meier survival curves illustrating the association of serum C-reactive protein (CRP) (≥10mg/L or <10 mg/L) and haptoglobin (<1.4 g/L or ≥1.4 g/L) levels with ovarian cancer patient survival in the AMORIS cohort (n = 177 patient sera). (e) Heatmap illustrating the patient survival when high expression of mediators involved in immune activation (Th1, Th2, Th17, M1, M2 and anti-parasitic/infection clearance), that are independently associated with better survival, are combined with high expression of inflammation and angiogenesis mediators that are independently associated with the worst prognosis (n = 30 combinations). (*P*-values: * = *P*< 0.05, ** = *P*< 0.01, *** = *P*< 0.001, **** = *P*< 0.0001).

We next considered whether mediators in inflammation and angiogenesis may be associated with clinical prognosis in ovarian cancer. Of the 13 markers evaluated in this category, independently elevated expression of CXCL-12 (HR = 1.48, *P*= 1.4x10^−7^), C-Reactive Protein (CRP) (HR = 1.25, *P*= 0.0049) and Platelet Derived Growth Factor subunit A (PDGFA) (HR = 1.19, *P*= 0.032) were significantly associated with negative prognostic outcomes in patients; the mediators were associated with increased risk of death by 48%, 25%, and 19%, respectively (Figure 6(a), Supplementary Figure 4).

Furthermore, high expression of 16.5% of the 1,092 combinations of mediators in this immune category was significantly associated with worse patient prognosis (52.3% were associated with HR>1 without statistical significance), while 1.4% were significantly correlated with positive prognosis (Figure 6(b)). CXCL-12 was the mediator most significantly associated with worse clinical outcome alone and in combination with other inflammation markers (Figure 6(c)).

### Inflammatory markers in patient sera show association with ovarian cancer-specific death

We next considered whether markers of inflammation measured in the circulation were also associated with an increased risk of death from ovarian cancer using the AMORIS cohort. Characteristics of study participants are shown in Table 1. During a mean follow-up of 6.5 years, 177 out of 289 women diagnosed with ovarian cancer died specifically from ovarian cancer. The mean value of CRP in serum was higher in women who died from ovarian cancer (11.67 mg/L) (n = 177) compared with women who did not (7.11 mg/L) (n = 112) (Table 1). Cox proportional hazards regression analysis showed a positive association with risk of dying from ovarian cancer for those with higher levels of serum haptoglobin (≥1.4 g/L) compared to those with haptoglobin levels <1.4 g/L (HR = 2.09, 95% CI:1.38–3.16) (Figure 6(d), Table 2). The median survival time for ovarian cancer was 1.3 years shorter when women had haptoglobin levels ≥1.4 g/L compared to women with haptoglobin levels <1.4 g/L. After a follow-up time of 20 years, 15% of the women with haptoglobin levels ≥1.4 g/L had survived ovarian cancer compared to 39% of the women with haptoglobin levels <1.4 g/L (log Rank P-value <0.001). No significant associations were observed with serum CRP, albumin or leukocytes (Figure 6(d), Table 2). Collectively, these findings may suggest that elevated cancer-associated inflammation markers, in the tumor and patient circulation, may have negative associations with patient survival in ovarian cancer.

**Table 1.** Descriptive statistics of study population from the AMORIS cohort.

	Ovarian cancer-specific death N = 177	No deaths specific to ovarian cancer N = 112
Mean Age, years (±SD)	61.4 (±11.81)	57.7 (±11.40)
Mean time between sampling and diagnosis, months (±SD)	24.5 (±18.55)	27.1 (±18.53)
Mean follow-up time, years (±SD)	2.7 (±3.06)	12.5 (±6.23)
**C-reactive protein (mg/L)**		
Mean, mg/L (±SD)	11.67 (±20.41)	7.11 (±10.22)
<10 mg/L, n (%)	139 (78.5%)	94 (83.9%)
≥10 mg/L, n (%)	38 (21.5%)	18 (16.1%)
**Albumin (g/L)**		
Mean, g/L (±SD)	41.54 (±3.03)	41.76 (±3.02)
<40 g/L, n %)	35 (19.8%)	20 (17.9%)
≥40 g/L, n (%)	136 (76.8%)	86 (76.8%)
Missing, n (%)	6 (3.4%)	6 (5.4%)
**Leukocytes (x10^9^ cells/L)**		
Mean, x10^9^ cells/L (±SD)	6.80 (±1.86)	6.99 (±2.02)
<10 × 10^9^ cells/L, n (%)	85 (48.0%)	58 (51.8%)
≥10 × 10^9^ cells/L, n (%)	4 (2.3%)	4 (3.6%)
Missing, n (%)	88 (49.7%)	50 (44.6%)
**Haptoglobin (g/L)**		
Mean, g/L (±SD)	1.31 (±0.55)	1.12 (±0.37)
<1.4 g/L, n (%)	72 (40.7%)	59 (52.7%)
≥1.4 g/L, n (%)	34 (19.2%)	11 (9.8%)
Missing, n (%)	71 (40.1%)	42 (37.5%)

**Table 2.** Age-adjusted hazard ratio (HR) for risk of ovarian cancer-specific death in the AMORIS cohort with 95% confidence intervals (CI) using Cox proportional hazards model.

	Hazard Ratio (95% Cl)
**C-reactive protein (mg/L)**	
<10 mg/L	1.00 (Ref)
≥10 mg/L	1.18 (0.82–1.69)
**Albumin (g/L)**	
<40 g/L	1.10 (0.75–1.61)
≥40 g/L	1.00 (Ref)
**Leukocytes (x10^9^ cells/L)**	
<10 × 10^9^ cells/L	1.00 (Ref)
≥10 × 10^9^ cells/L	0.88 (0.32–2.41)
**Haptoglobin (g/L)**	
<1.4 g/L	1.00 (Ref)
≥1.4 g/L	2.09 (1.38–3.16)

### Higher intratumoral expression of immune activation markers may rescue or neutralize the negative associations of inflammation or angiogenesis

Based on the differences in patient survival associated with mediators of immune activation compared to those of inflammation and angiogenesis, we sought to investigate whether intratumoral signatures of classical, anti-parasitic/infection and M1/M2-type macrophage immune responses could influence the associations of inflammatory and angiogenesis signatures with worse clinical prognosis. Mediators independently associated with better survival were selected from each immune category (namely CXCL-13, IL-23, CXCL-9, CXCL-10, CXCL-11, TNFα, IFNγ, CCL-7, MCP-1, and IL-4) and combined with each of the three inflammation and angiogenesis mediators associated with the worst patient survival (CXCL-12, CRP, PDGFA) (n = 43 tests, of which n = 30 were combinations, Figure 6(e)). Combination of high expression of any of the classical, anti-parasitic or macrophage immunity mediators with PDGFA and/or CRP was associated with either reduction of HR or reversal towards better prognosis (Figure 6(e)). This effect was particularly apparent when Th1/M1 mediators, CXCL-9, CXCL-10, and CXCL-11, were included. On the other hand, high expression of CXCL-12, combined with any classical immune mediator, continued to retain a negative association with patient survival, however, combination with high CXCL-9 and CXCL-10 expression, appeared to partly “neutralize” its negative prognostic association.

Overall, these suggest that combined high intratumoral expression of mediators of classical immune responses, alongside inflammation signatures otherwise associated with poor prognosis, may moderate, reduce or reverse the negative associations of inflammation and angiogenic mediators with patient survival.

## Discussion

We investigated immune and inflammatory mediator expression signatures and their clinical significance in ovarian cancer. We identified 44 mediators known to be involved in immune response to pathogens and in inflammation. In 954 samples, including 839 ovarian cancer tissues and 115 non-malignant ovarian tissue samples, we found that gene expression of the 44 mediators could differentiate between ovarian cancer and non-malignant tissues with 96–98% accuracy, by machine learning methods. By evaluating immune and inflammatory mediator gene expression profiles from 1,656 ovarian carcinoma patient tumors in silico, we demonstrated that higher genomic expression of some classical Th1, Th2, Th17, anti-parasitic/infection and M1-macrophage associated secreted mediator signatures were associated with better clinical outcomes. Contrastingly, we found negative associations of the intra-tumoral genomic expression of inflammatory and angiogenic markers with ovarian cancer patient OS. In concordance, the inflammation marker haptoglobin in the sera of 289 ovarian cancer patients from the AMORIS cohort showed a negative association with survival. Our study therefore highlights links between immune protection and inflammation mediator signatures with ovarian cancer outcomes.

The association of immune-related gene signatures to disease subgroups is critical in understanding disease mechanisms and prognosis.^25^ Supervised learning such as machine learning methods employed here, are increasingly popular means of computational classification of cancer samples into appropriate phenotypes and ranking the discriminatory power of each gene in the disease classification outcome.^26^ When machine learning was used to discriminate between non-malignant and cancerous samples, excellent prediction accuracy was reported when the set of 44 selected genes was used. Through LDA, the ovarian cancer samples clearly separated from non-malignant tissues, illustrating that the selected 44 mediators are efficient discriminatory features. These indicate that the selected genes (features) are able to classify tissue types and supports the importance of these published literature-selected markers.

Elevated intratumoral expression of Th1 immune mediators, CXCL-9, CXCL-10 and CXCL-11 – which share the same receptor, CXCR3 – were associated with better prognosis. Consistent with our findings, the CXCL-9/CXCL-10/CXCL-11–CXCR3 axis has been reported to lead to enhanced tumor growth restriction by recruitment of CD8 + T cells (CTLs), NK cells and macrophages.^27–31^ In mouse models, CXCL-11, as an adjuvant to oncolytic virus-based treatments, could significantly enhance their anti-tumor efficacy,^29^ and CXCL-11-Fc fusions enhanced recruitment of antigen-specific CD8 + T cells.^32^ Furthermore, secretion of these three mediators by monocytes, endothelial cells, fibroblasts and cancer cells was enhanced through transcriptional activation of STAT1 and NFκB, caused by IFNγ and TNFα working in synergy.^33^ In our study, elevated expression of both IFNγ and TNFα were also associated with better 5-year OS, pointing to putative anti-tumor functions.

We observed that IL-4, a key Th2-secreted cytokine that mediates lymphocyte differentiation and orchestrates allergic diseases, was significantly associated with better prognosis. Although IL-4 has been linked with pro-tumor properties,^34–36^ mice overexpressing IL-4 showed significant tumor growth reduction compared to non-transgenic mice,^37^ and there are reports of inverse correlations between allergic conditions and the risk of developing certain types of cancer including ovarian cancer.^38–40^ Here, the positive prognostic association of IL-4 and some Th2 signatures may suggest potential anti-tumor functions in ovarian carcinomas.

Both IL-23 and CXCL-13 may facilitate pro-tumor phenotypes through initiating tumorigenesis, increasing angiogenesis,^41^ promoting metastasis^42^ and reducing infiltration of CD8 + T cells^43^ in colorectal and non-small cell lung carcinoma (NSCLC), and by maintaining self-renewability of ovarian cancer stem cells (OCSCs) within the tumor microenvironment.^44^ In our analyses, we found a significant correlation between higher expression of the Th17 differentiation cytokine IL-23 with better OS, in line with a study by Wolf et al.^45^ We also found that higher expression of the chemokine CXCL-13 was also associated with better OS in our cohort. There are no reports with regards to beneficial effects of CXCL-13 in ovarian cancer, however, links with improved outcome have been observed in HER2+ breast cancer patients.^46^ Combined with results of our study these associations suggest possible anti-tumor rather than tumor-promoting roles for IL-23 and CXCL-13 in the ovarian cancer context and warrant further investigation.

We also found positive prognostic associations with elevated levels of TNFα and MCP-1, immune mediators involved in Th1 and Th2 immunity, respectively, but also in anti-parasitic/infection responses. MCP-1, a potent monocyte chemoattractant can support anti-parasitic immunity, enhancing monocyte/macrophage and cytotoxic T cell migration and retention into infected tissues, and facilitating parasite uptake and destruction by macrophages.^47^ MCP-1 may recruit classical monocytes from nearby blood vessels that may differentiate into immune-activating M1 macrophages, which may suppress tumor growth and development.^48^ We recently identified an IgE-potentiated TNFα/MCP-1 axis associated with tumor growth restriction mediated by activated macrophages in vivo.^11,12,49^ Here, our analyses in ovarian cancer tumor samples also indicate that a TNFα/MCP-1 signature could have a role in anti-cancer immunity. This and other immune signatures normally deployed in infection clearance may potentially be enhanced by specific immunotherapy approaches such as a tumor-specific IgE or by attenuated parasite vaccines,^50,51^ to reverse immunosuppression and confer therapeutic benefits in ovarian and other cancers.

A broad spectrum of activation states is exhibited by tumor-associated macrophages (TAMs); the two extremes of which are classically activated M1 and alternatively activated M2 phenotypes.^52^ Our analyses indicated that the majority of M1-type macrophage mediators were associated with improved prognosis, while only 2% of M2-type markers significantly correlated with better survival. These likely reflect distinct roles of classically- and alternatively activated macrophages within the tumor microenvironment. M1-type macrophages have both direct and indirect tumoricidal effects: direct effects by release of reactive oxygen and nitrogen intermediates, leading to tumor cell cytotoxicity and anti-proliferative effects;^53^ and indirect effects by enhancing T cell- and NK-driven anti-tumor immunity through secreting cytokines such as CXCL-9, CXCL-10, CXCL-11, IL-23 and TNFα. M1-produced factors thus establish a highly cytotoxic, tumor-rejecting environment.^53^ By contrast, alternatively activated M2-type macrophages may fail to elicit potent immune responses against cancerous cells, leading to neovascularization, cancer spread and local suppression of innate and adaptive anti-tumor immune responses.^54^ M2 mediators such as TGF-β, CCL-17 and CCL-22 may inhibit Th lymphocytes, CTLs, NK cells, macrophages, B cells and antibody synthesis, and support cancer cell proliferation and tumor angiogenesis.^55,56^ Despite these proposed pro-tumoral functions, our study demonstrates that over 97% of the M2 combinations were not associated with patient survival outcomes, perhaps pointing to tolerance to rather than promotion of tumor growth. These may suggest that re-education of macrophages toward an M1-like functional phenotype might offer therapeutic advances.^10,49,57,58^

Inflammation has been linked to the risk of cancer development in a range of malignancies. Components of inflammation have been reported to contribute to proliferation, migration and survival of ovarian cancer cells through mutations, genomic instability and epigenetic modifications, or by stimulating tissue repair, angiogenesis and causing localized immunosuppression (Supplementary Table 1). However, inflammatory cells and molecules can also drive effective anti-cancer immunity capable of eradicating cancer or delaying its development.^59^ In our Random Forest analysis, VEGF and PDGFA were of the highest importance among the 44 selected genes in discriminating cancer from non-malignant ovary tissue and elevated CXCL-12, CRP and PDGFA, were significantly associated with worse prognosis in our study of intratumoral expression. CXCL-12, likely secreted by cancer-associated fibroblasts (CAFs), may promote accumulation of FoxP3 + T cells and cancer cell survival in high grade serous ovarian cancer.^60^ Moreover, binding of CXCL-12 to its receptor CXCR4 has been shown to induce tumor proliferation and cancer progression, and these effects were attenuated when this pathway was knocked down in pre-clinical in vivo models.^61^ PDGFA is a potent activator for mesenchymal-origin cells, and stimulates chemotaxis, proliferation and angiogenesis in cancer,^62^ engaging several pathways such as Ras-MAPK, PI3K, PLCγ and inducing VEGF production.^62,63^ Plasma CRP concentration is increased in response to inflammation, tissue damage and infection, and may promote tumor growth and metastasis by causing low-grade inflammation in breast cancer, although the exact pathogenesis is still uncertain.^64,65^ In our analyses, we report a significant association between high intratumoral CRP expression with poor patient prognosis. Comparably, in the Swedish AMORIS cohort, during a mean follow-up of 6.5 years, the mean serum CRP levels measured around the time of diagnosis were higher in women who subsequently died from ovarian cancer. This is consistent with previous reports of elevated serum CRP associated with a higher risk of developing ovarian cancer.^66^ Furthermore, increased serum levels of the inflammation marker haptoglobin was significantly associated with poorer survival, aligning with reports pointing to haptoglobin as a potential serum biomarker in ovarian cancer.^67,68^ Although the mechanism of how haptoglobin contributes to cancer progression is not yet clear, evidence points to contributions to ovarian cancer cell migration, through cell morphology and alterations in actin cytoskeleton.^69^ Based on analyses of three cohorts, our findings therefore point to significant roles of inflammation and angiogenesis mediators in ovarian cancer patient circulation and tumors.

Independently, high intratumoral expression of the Th1 cytokine TNFβ and of the anti-parasitic mediator CCL-21 was significantly associated with worse prognosis. When combined with high expression of other cytokines in the same immune category, we noted reductions in HR (HR<1). Furthermore, combined mediators of immune activation (which individually were associated with positive prognosis) with markers of inflammation and angiogenesis (which were independently associated with worse survival), could neutralize or reverse some of the negative prognostic associations of inflammation and angiogenic markers. From these observations, it is tempting to speculate that potential therapeutic approaches able to activate specific immune signatures or reverse immunosuppression may be desirable.

Taken together, our analyses integrating: (a) selection of 44 secreted mediators involved in immune responses and inflammation through literature search, (b) gene expression by machine learning methods in 954 ovarian cancer and non-malignant ovary tissues, (c) 1,978 immune and inflammatory mediator signatures from genomic expression data of 1,656 ovarian carcinoma patient tumors, and (d) 289 sera from patients with ovarian cancer in the AMORIS cohort study, all point to the significance of immune secretomes for clinical outcomes. Immune activation, whether classical, anti-parasitic, or supporting macrophage re-education to classically activated phenotypes, may provide an immunological advantage in ovarian cancer, and inflammatory and angiogenic signatures may contrastingly confer negative effects for prognosis. Even the negatively prognostic elevated expression of inflammatory or angiogenic mediators may be “neutralized” when expressed along with beneficial secretome states and could be taken into consideration in immunomonitoring and when developing or selecting therapies.^20^ Future work could focus on the roles of specific immunological states in patient cohorts, including different malignant histologies, or stratification of treatment-naïve, chemotherapy or immunotherapy pre-treated patients. These may help identify common secretome algorithms linked to improved survival. Insight into these interactions and immune signatures can facilitate the development of novel tailored immunotherapies that seek to enhance beneficial host immunity.

## Materials and methods

### Selection of immune cell markers for analysis

We performed an extensive literature review focusing on secreted immune mediators associated with different categories of immune activation with reported possible roles in cancer immunity and we identified 44 immune mediator markers (cytokines, chemokines and soluble inflammation and angiogenic factors). These 44 markers were categorized into the following groups: Th1, Th2, Th9, Th17, M1 macrophage, M2 macrophage, anti-parasitic/infection response, and, angiogenesis and inflammation (Figure 1, Supplementary Table 1).

### Microarray gene expression data analysis for malignant disease classification

A dataset of 954 samples that includes 839 ovarian cancer tissues and 115 non-malignant samples was obtained from Gene Expression Omnibus (https://www.ncbi.nlm.nih.gov/geo/) Affymetrix Platform GPL570 (HG-U133 Plus 2.0) (GSE_IDs from previous reports).^70^ The microarray data of Affymetrix chips consists of a series of CEL files containing raw intensities for each probe on the array. Probe intensity was normalized by applying the R BioConductor RMA (Robust Multichip Average) function.^71^ Probes were mapped to genes using Affymetrix Human Genome U133 Plus 2.0 Array notation data (chip HG-U133 Plus 2) and in cases where multiple probes were mapped to the same gene, probe intensities were averaged.

### Assessing ability of selected 44 markers to differentiate between ovarian cancer and non-malignant tissue through machine learning

Three popular machine learning methods, Support Vector Machine, Random Forest, and Neural Networks were applied to classify ovarian cancer and non-malignant tissue samples using either all the genes available from the database, or just the 44 mediator genes as prediction features. These models were implemented using Python Scikit-Learn library. All the experiments were run on a workstation with a 12-core Intel Core i7 and 32GB of memory running Ubuntu Linux 16.04. For Support Vector Machine, we used Scikit-Learn svm.SVC function with “linear” kernel and 1.0 penalty error parameter (C). Similarly, RandomForestClassifier with 900 trees in the forest (n_estimator parameters) and MLPClassifier for the implementation of neural networks with two layers of size 800 and 100, “adam” solver, 0.01 initial learning rate, and 1e-5 regularization parameter (“alpha”) were used. After the random forest classifier was trained, “feature_importances_attribute” was used to determine the importance of each feature in the classification.

Linear Discriminant Analysis (LDA) was used to map high-dimensional input data into a two-dimensional space and visualize them. Visualization is a common way of summarizing data to show the ability of the input features to represent the labels. LDA extracts a linear combination of input features that distinguish the tissue type classes.^72^ In terms of the cross-validation scheme of the machine learning experiment, we performed 5 times 10 fold cross validation, where samples (954) were split into two sets of training and test sets (90/10 data split). Since the gene expression data have a large ratio of number of features vs. number of samples, we performed oversampling on our imbalanced dataset by applying SMOTE (Synthetic Minority Over-sampling Technique, SMOTE function in imbalanced-learn 0.4.2 Python package) on the training dataset to avoid overfitting.^73^ The model is built on the training set and then accuracy is assessed on the test set. In terms of ranking the importance of the 44 gene features, the Random Forest mean decrease in accuracy is used.^74^

### In silico prognostic association assessments of mediator expression signatures

This study capitalizes on publicly available datasets accessed through a publicly available online survival analysis tool. The KM-Plotter platform was employed in order to interrogate survival-associated biomarkers in ovarian cancer using microarray data and assess the impact of single immune markers and marker combinations on patient survival in ovarian cancer (Link to KM-Plotter platform http://kmplot.com/analysis/index.php?p=service&cancer=ovar).^75^ Available data encompassed Gene Expression Omnibus (GEO) and The Cancer Genome Atlas (TCGA) datasets, which provided microarray-generated intratumoral gene expression information from Affymetrix platforms GPL96 (Affymetrix HG-U133A), GPL570 (Affymetrix HG-U133 Plus 2.0) and GPL571/GPL3921 (Affymetrix HG-U133A 2.0). These are linked with survival data for 13,435 markers and tumor specimens from 1,656 ovarian carcinoma patients linked with overall survival (OS) data, updated from 1,287 samples included in the original report.^75^ Only publications with available raw data, clinical survival information and at least 20 patients were included in the cohort.^75^ Most tissue samples were collected from patients with advanced ovarian cancers who had undergone surgical debulking, a procedure commonly used to treat ovarian tumors.^76^

Patients were stratified into two risk groups based on expression levels of the selected mediator or of the combination of two, three, or four mediators; one group included subjects with the top 25% of expression of the mediator(s), whilst the second group included all other patients with lower expression in their tumors. The effects of differential expression on overall patient survival were assessed by Kaplan Meier-plots displaying the proportion of individuals surviving in each cohort over a course of 5 years. Hazard ratios (HR), 95% confidence intervals and *P*-values (<0.05 considered to be statistically significant) were calculated for all interrogated signatures. Optimal probe sets were filtered by the JetSet method which allowed selection of the most representative probe sets for each gene and was used for all evaluations to select for specificity, coverage and degradation resistance of probes and enhance consistency and maximize data accuracy.^75^ Batch effects were accounted for through a double normalization of microarray chip-derived data: a MAS5 algorithm-based normalization on individual-chip level followed by scaling normalization to set the average expression on each chip to 1,000. Analyses in the KM-Plotter accounted for censored data, including losses from the patient cohort over the follow-up threshold. Due to the exploratory nature of our analysis and in order to avoid increasing type II error (false negatives), results reported by KM plotter are reported as provided by the KM plotter resource without correction for multiple testing.^77^

A total of 1,978 single and combinations of secreted mediators were investigated derived from the 44 genes: 231 tests where two mediators were combined, 591 tests where three mediators were combined, and 1,112 tests where four mediators were combined. All possible combinations of up to four were investigated within each immunity category (Supplementary Figure 1). In order to investigate the impact of each combination of mediator expression, we employed the “multigene classifier” and “mean expression of selected probes” functions provided by the KM plotter. This enabled us to create a signature which is the mean expression of selected genes, e.g. expression of gene (A + B + C)/3 = signature; which was then used to assess the impact on overall survival.

### Inflammation markers in sera of patients with ovarian cancer in the AMORIS database

#### Study population and data collection

The Swedish Apolipoprotein MOrtality-related RISk (AMORIS) cohort consists of participants predominantly living in greater Stockholm in Sweden during the baseline period 1985–1996. At the time of inclusion, all participants were either healthy individuals referred for a health check-up by their employers or were outpatients. Between 1985 and 1996, over 500 different blood biomarkers had been measured in 812,073 individuals. The blood samples were analyzed at the Central Automation Laboratory (CALAB) in Stockholm. With the use of a 10-digit personal identification code used for all residents of Sweden, the AMORIS cohort was linked with a variety of national registers. Of particular importance for the present study, this linkage included the Swedish national cancer register and cause of death register, respectively. This enabled a basically complete follow-up of incident (new) cases of cancer and cause specific death until the end of follow-up in 2011. Additional national registers provide data on education, socio-economic status, comorbidities and emigration. The AMORIS cohort and its linkages has been described in a cohort paper recently published.^21^ The current study conformed to the declaration of Helsinki and was approved by the ethics board of Karolinska Institutet.

We included all individuals who had a diagnosis of ovarian cancer (International Classification of Disease (ICD), Revision 7 (1995) code 175) and serum CRP measured between 6 days and 5 years before the diagnosis of ovarian cancer (n = 289). Measurements of serum albumin (n = 277), haptoglobin (n = 176) and white blood cells (n = 151) were also taken into account when available. Follow-up time was defined as the time from date of ovarian cancer diagnosis until date of death or end of the study (31^st^ December 2011), whichever occurred first. The outcome evaluated was ovarian cancer-specific death (ICD 9/10 code 183, C56). We included the following information from the AMORIS study: serum CRP (mg/L), albumin (g/L), leukocytes (x10^9^ cells/L), haptoglobin (g/L) and age at ovarian cancer diagnosis.

The quantitative determination of serum CRP and haptoglobin was measured with an immunoturbi-dimetric assay (reagents from Orion Diagnostics, Espoo, Finland) using fully automated multichannel analyses (for CRP an AutoChemist – PRISMA, New Clinicon, Stockholm, Sweden 1985–1992 and a DAX 96, Technicon Instruments, Corporation, Tarrytown, NY, USA, 1993–1996; for haptoglobin Hitachi-analysers, Boehringer Mannheim, Baden-Wurttemberg, Germany). High sensitivity CRP was not available during the period of blood sample collection (1985–1996). Therefore, CRP < 10 mg/L could not be measured precisely. However, the 10 mg/L cut-off has been widely accepted as the upper limit of the health-associated reference range. Albumin was measured using the bromocresol green method. Leukocytes were measured by routinely used hematology analyzers (STKS Haematology System from Coulter Corporation, Hialeah, FL). Total imprecision calculated by the coefficient of variation was 12% at CRP level 40 mg/L, 5.6% at haptoglobin level 1.1 g/L, <1.8% for albumin and <2.7% at leukocytes 10 × 10^9^ cells/L.^78^

#### Statistical analysis

We estimated the risk of ovarian cancer-specific death with Cox proportional hazards regression for medical cut-offs used in the CALAB laboratory for CRP: <10 mg/L and ≥10 mg/L; haptoglobin: <1.4 g/L and ≥1.4 g/L; leukocytes: <10x10^9^ cells/L and ≥10x10^9^ cells/L.^79^ Albumin was dichotomized as <40 g/L and ≥40 g/L instead of the medical cut-off of 35 g/L due to the small number of participants with low albumin levels.^80^ All models were adjusted for age. We additionally created a Kaplan–Meier curve to graphically represent those associations observed to be statistically significant in the Cox models. All statistical analyses were conducted with Statistical Analysis Systems (SAS) release 9.4 (SAS Institute, Cary, NC).
