# Supplementary material for: Immune mediator expression signatures are associated with improved outcome in ovarian carcinoma
Source: Oncoimmunology. 2019 Mar 28;8(6):e1593811. doi: 10.1080/2162402X.2019.1593811 (PMC6492968; doi:10.1080/2162402X.2019.1593811)
Supplement: Supplemental Material [file koni-08-06-1593811-s001.zip › Supplementary Figure 1.pptx]

## Slide 1
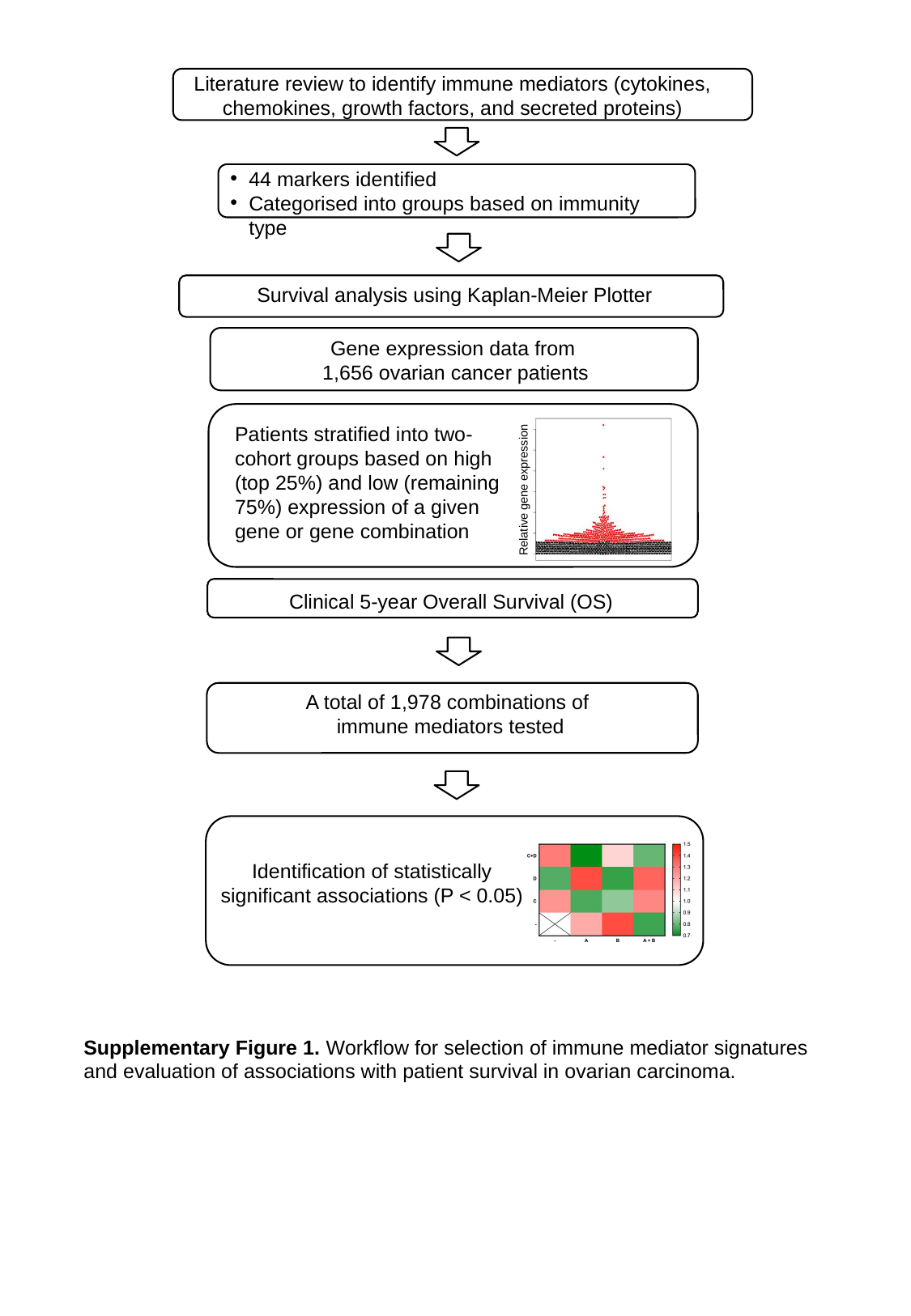

Literature review to identify immune mediators (cytokines, chemokines, growth factors, and secreted proteins)
44 markers identified
Categorised into groups based on immunity type
Survival analysis using Kaplan-Meier Plotter
Gene expression data from
1,656 ovarian cancer patients
Patients stratified into two-cohort groups based on high (top 25%) and low (remaining 75%) expression of a given gene or gene combination
Relative gene expression
Clinical 5-year Overall Survival (OS)
A total of 1,978 combinations of
immune mediators tested
Identification of statistically significant associations (P < 0.05)
Supplementary Figure 1. Workflow for selection of immune mediator signatures and evaluation of associations with patient survival in ovarian carcinoma.
