# Supplementary material for: Immune mediator expression signatures are associated with improved outcome in ovarian carcinoma
Source: Oncoimmunology. 2019 Mar 28;8(6):e1593811. doi: 10.1080/2162402X.2019.1593811 (PMC6492968; doi:10.1080/2162402X.2019.1593811)
Supplement: Supplemental Material [file koni-08-06-1593811-s001.zip › Supplementary Table 1.docx]

**Supplementary Table 1.** Secretome mediators (n = 44) selected for analysis to investigate associations of immune response signatures with ovarian cancer patient survival.

| **Secretome mediator** | **Immune category** | **Function in cancer** | **Study model** |
| --- | --- | --- | --- |
| CCL-3 /MIP-1α* | Anti-parasitic/infection [1-4] | Promotes tumour immune suppression by enhancing retention of MAMs* [5], CAFs* and enhancing cell migration [6] | Human [2-4]  Mouse [5, 6] |
| CCL-4/ MIP-1β* | Anti-parasitic/infection [1-4] | Promotes tumour immune suppression by assisting bone metastasis [7] and Treg infiltration [8], but also recruits CD8+ T cells [9] | Human [2-4, 9]  Mouse [7, 8] |
| CCL-5/ RANTES | Anti-parasitic/infection [1, 3, 4] | Promotes tumour immune suppression by assisting Treg infiltration [8] and cancer cell metastasis/migration [10] | Human [3, 4, 10]  Mouse [8] |
| CCL-7 /MCP-3 | Anti-parasitic/infection [1, 11-14] | Promotes cancer cell migration and metastasis [15-17], but can trigger anti-tumour response through recruitment of activated NK and monocytic cells [18, 19] | Human [12, 14-16]  Mouse [11, 13, 17-20] |
| CCL-8 /MCP-2 | Anti-parasitic/infection [1, 11, 12] | Promotes metastasis [21] and Treg accumulation [22], but also inhibits tumour cell migration [23] | Human [12, 21, 23]  Mouse [11, 22, 23] |
| CCL-17 | M2 [24, 25] | Treg accumulation [26] | Human [24, 26] |
| CCL-18 | M2 [25] | Promotes cancer cell migration and metastasis [27, 28] | Human [27, 28]  Mouse [28] |
| CCL-19 | Anti-parasitic/infection [1, 29] | Promotes tumour invasion [30, 31], but also prevents tumour progression by assisting mononuclear infiltration [32, 33] | Human [31]  Mouse [29, 32]  Cell lines [30]  3D in vitro model [33] |
| CCL-21 | Anti-parasitic/infection [1, 29] | Promotes cellular migration and metastasis [34], and lymphangiogenesis [35] | Human [34, 35]  Mouse [29, 35] |
| CCL-22 | M2 [24, 25] | Recruits Tregs into tumour microenvironment, and inhibits T cell activation promoting tumour growth and metastasis [26, 36, 37] | Human [24, 26, 36, 37]  Mouse [36, 37] |
| CCL-24 | M2 [25] | Promotes angiogenesis [38] | Human and mouse [38] |
| CRP* | Inflammation & angiogenesis [39, 40] | Promotes tumour growth and metastasis [39], and reduces cancer cell apoptosis [41] | Human and mouse [41] |
| CXCL-9 | Th1 [25, 42]/ M1 [25] | Suppresses tumour growth by TIL recruitment [43, 44], and inducing CD8+ T-cell mediated functions [45] | Human [43]  Mouse [44, 45] |
| CXCL-10 | Th1 [25, 42]/ M1 [25] | Suppresses tumour growth by TIL recruitment [43] | Human [43] |
| CXCL-11 | Th1 [42, 46, 47]/ M1 [25] | Promotes anti-tumour immunity by enhancing CD8+ T cell response and recruitment [46-48] | Mouse [46-48] |
| CXCL-12 | Inflammation & angiogenesis [49] | Enhances survival of tumour CAFs* [50] and accumulation of Foxp3+ T cells [50, 51] | Human [49-51]  Mouse [49, 51] |
| CXCL-13 | Th17 [52] | Promotes tumour metastasis [53] and EMT* [54]  Associated with improved clinical outcome in breast cancer [55] | Human [52-55] |
| GM-CSF | Th1 [56, 57] | Promotes intra-tumoural immunosuppression, vascularization and local/metastatic cancer progression [58, 59]  Inhibits cancer cell growth and associated with improved survival [60] | Human [56, 60] Mouse [57-59] |
| HIF1α | Inflammation & angiogenesis [61, 62] | Contributes in metabolic reprogramming of cancer cells in favour of tumour survival [63, 64] | Human [63]  Mouse [63, 64] |
| IFNγ | Th1 [65, 66] | Classically known to exhibit anti-tumour activity by recruiting CTLs*, promoting cancer cell apoptosis, and preventing angiogenesis [67] and proliferation [20, 68] | Human [20, 68]  Mouse [20, 67, 68] |
| IL-1β | M1 [24, 25]/ Inflammation & angiogenesis [69-71] | Promotes angiogenesis via recruitment of VEGFR1* positive cells [71]  Exerts strong inflammatory environment [72, 73] | Human [72, 73] Mouse [71-73] |
| IL-2 | Th1[65, 66] | Promotes anti-tumour immunity by expanding, activating and enhancing activities of cytolytic cells [74-76] | Human [74]  Mouse [75, 76] |
| IL-3 | Th2 [77]/ Inflammation & angiogenesis [78] | Promotes angiogenesis [78] and cell growth/proliferation [79-81]  Reports claim anti-angiogenic properties [82] | Human [81, 82]  Mouse [78]  Cell lines [79, 80] |
| IL-4 | Th2 [65, 66]/ M2 [83-85] | Often reported as pro-tumour but also contributes to anti-tumour immunity by inducing tumour cell apoptosis [86, 87], and recruiting of immune effector cells [88-90] | Mouse [88-90]  Cell lines [86] |
| IL-5 | Th2 [65, 66] | Promotes tumour migration, invasion and metastasis [91], [92] | Human [91]  Mouse [92] |
| IL-6 | M1 [25]/ Inflammation & angiogenesis [93, 94] | Promotes tumour growth by hyperactivating JAK/STAT3 pathway which down modulates anti-tumour immunity [95-97], and positively regulates Tregs and MDSCs* [95, 98] | Mouse [95-97] |
| IL-8 | Inflammation & angiogenesis [99] | Promotes angiogenesis and metastasis [100-103] | Mouse [100-103] |
| IL-9 | Th9 [104, 105] | Increases leukocyte infiltration and CD8+ T cell-mediated cytotoxicity [104, 105] | Human [106]  Mouse [104] |
| IL-10 | Th2 [65, 66]/ M2 [107] | Classically known as pro-tumour by being immunosuppressive, but also recently reported to have anti-tumour activities by activating CD8+ T cells [108-110] | Mouse [108, 110] |
| IL-12 | Th1 [111, 112]/ M1 [25] | Promotes proliferation and maturation of cytolytic immune effector cells [111-113]  Prevents angiogenesis [114] | Human [111, 112]  Mouse [113, 114] |
| IL-13 | Th2 [65, 66] | Promotes tumour invasion and metastasis [115-117] | Mouse [115-117] |
| IL-17 | Th17 [118-120] | Enhances tumour growth via γδ T cells [121, 122] | Human [118]  Mouse [121, 122] |
| IL-18 | Inflammation & angiogenesis [123] | Promotes tumour growth through accumulation of MDSCs [124], but also promotes anti-tumour immunity by suppression of Tregs, and recruitment/enhancement of NK and CD8+ T cells [123, 125] | Human [123]  Mouse [123-125] |
| IL-21 | Th17 [126] | Promotes anti-tumour immunity by counteracting Treg-mediated inhibition of [127, 128], and boosting responses of CD4 T cells [129] | Human [127, 128]  Mouse [126, 129] |
| IL-22 | Th17 [130] | Induced by cancer cells and produced by CD4+ T cells [73], and CAFs* [131] to promote tumour growth and invasion | Human [73, 131] Mouse [73, 130, 131] |
| IL-23 | Th17 [120]/ M1[132, 133] | Promotes tumour immunity by initiating tumourigenesis and progression [134, 135], increasing angiogenesis and reducing CD8+ T cell infiltration [136] | Human [134, 136]  Mouse [134-136] |
| IL-33 | Inflammation & angiogenesis [137-139] | Promotes tumour metastasis and growth by recruiting MDSCs* and lessening NK cell cytotoxicity [137, 140] and increasing transcriptional activity [138, 140] | Human [138, 140] Mouse [137-140] |
| IL-36 | Inflammation & angiogenesis [141, 142] | Suppresses tumour growth by promoting IFNγ production by CD8, NK, γδ T cells [142] | Human [142]  Mouse [142] |
| MCP-1 /CCL-2 | Anti-parasitic/infection [1, 3, 143]/ Th2 [144] | Promotes tumour immunity by enhancing retention of MAMs* [5], also suppresses tumour growth by recruiting tumour-associated macrophages [143] | Human [3]  Mouse [5, 143, 144] |
| PDGFA | Inflammation & angiogenesis [145, 146] | Promotes tumour immunity by assisting angiogenesis by promoting VEGF* production [146], and increasing proliferation and invasiveness of cancer [147] | Human [146]  Mouse [147] |
| TGFβ | Th2 [148, 149]/ M2 [150, 151]/ Inflammation & angiogenesis [152-154] | Promotes tumour progression by assisting angiogenesis [153, 154] and cell invasiveness [155] | Human [151]  Mouse [148, 149, 151, 154, 155]  Cell lines [153] |
| TNFα | Anti-parasitic/infection [156-158]/ Th1 [66]/ M1 [25] | Promotes anti-tumour immunity by increasing infiltration of macrophages via TNFα-MCP-1 axis [159], inducing cytotoxic activities by CTLs* [160] and NK cells [161] | Human [159]  Mouse [159, 160]  Rat [159]  Cell Lines [161] |
| TNFβ/*LTα | Th1 [66] | Known to assist tumour growth, metastasis and angiogenesis through activating its receptor, LTβR* [162-164] | Mouse [162-164] |
| VEGF | Inflammation & angiogenesis [165, 166] | Promotes angiogenesis [165, 166], assists Treg proliferation at tumour site [167], and tumour cell growth through NRP-1* signaling [168] | Human [167]  Mouse [167, 168] |

* Macrophage inflammatory protein 1-alpha, MIP-1α; Macrophage inflammatory protein 1-beta, MIP-1β; Metastasis-associated macrophages, MAMs; Cancer-associated fibroblasts, CAFs; Epithelial mesenchymal transition, EMT; Myeloid-derived suppressor cells, MDSCs; Cytotoxic T lymphocytes, CTLs; Vascular endothelial growth factor, VEGF; Vascular endothelial growth factor receptor 1, VEGFR1; C-Reactive Protein, CRP; Lymphotoxin alpha, LTα; Lymphotoxin beta receptor, *LTβR;* Neuropilin 1, NRP-1;

**Supplementary References**

1. McGovern, K.E. and E.H. Wilson, *Role of Chemokines and Trafficking of Immune Cells in Parasitic Infections.* Curr Immunol Rev, 2013. **9**(3): p. 157-168.

2. Kocherscheidt, L., et al., *Cytokine and chemokine responses in adults, newborns and children exposed to Entamoeba histolytica/dispar, Onchocerca volvulus and Plasmodium falciparum.* Pediatr Allergy Immunol, 2010. **21**(4 Pt 2): p. e756-63.

3. Ritter, U., et al., *Differential expression of chemokines in patients with localized and diffuse cutaneous American leishmaniasis.* J Infect Dis, 1996. **173**(3): p. 699-709.

4. Cocchi, F., et al., *Identification of RANTES, MIP-1 alpha, and MIP-1 beta as the major HIV-suppressive factors produced by CD8+ T cells.* Science, 1995. **270**(5243): p. 1811-5.

5. Kitamura, T., et al., *CCL2-induced chemokine cascade promotes breast cancer metastasis by enhancing retention of metastasis-associated macrophages.* The Journal of Experimental Medicine, 2015. **212**(7): p. 1043-1059.

6. Tanabe, Y., et al., *Blockade of the chemokine receptor, CCR5, reduces the growth of orthotopically injected colon cancer cells via limiting cancer-associated fibroblast accumulation.* Oncotarget, 2016. **7**(30): p. 48335-48345.

7. Sasaki, S., et al., *Essential roles of the interaction between cancer cell-derived chemokine, CCL4, and intra-bone CCR5-expressing fibroblasts in breast cancer bone metastasis.* Cancer Lett, 2016. **378**(1): p. 23-32.

8. Schlecker, E., et al., *Tumor-Infiltrating Monocytic Myeloid-Derived Suppressor Cells Mediate CCR5-Dependent Recruitment of Regulatory T Cells Favoring Tumor Growth.* The Journal of Immunology, 2012. **189**(12): p. 5602-5611.

9. Liu, J.Y., et al., *CTL- vs Treg lymphocyte-attracting chemokines, CCL4 and CCL20, are strong reciprocal predictive markers for survival of patients with oesophageal squamous cell carcinoma.* Br J Cancer, 2015. **113**(5): p. 747-55.

10. Singh, S.K., et al., *CCR5/CCL5 axis interaction promotes migratory and invasiveness of pancreatic cancer cells.* Sci Rep, 2018. **8**(1): p. 1323.

11. Mennechet, F.J., et al., *Lamina propria CD4+ T lymphocytes synergize with murine intestinal epithelial cells to enhance proinflammatory response against an intracellular pathogen.* J Immunol, 2002. **168**(6): p. 2988-96.

12. Ruhwald, M., et al., *IP-10, MCP-1, MCP-2, MCP-3, and IL-1RA hold promise as biomarkers for infection with M. tuberculosis in a whole blood based T-cell assay.* BMC Res Notes, 2009. **2**: p. 19.

13. Jia, T., et al., *Additive roles for MCP-1 and MCP-3 in CCR2-mediated recruitment of inflammatory monocytes during Listeria monocytogenes infection.* J Immunol, 2008. **180**(10): p. 6846-53.

14. Carneiro, M.W., et al., *Gene Expression Profile of High IFN-gamma Producers Stimulated with Leishmania braziliensis Identifies Genes Associated with Cutaneous Leishmaniasis.* PLoS Negl Trop Dis, 2016. **10**(11): p. e0005116.

15. Hwang, T.L., et al., *CCL7 and CCL21 overexpression in gastric cancer is associated with lymph node metastasis and poor prognosis.* World J Gastroenterol, 2012. **18**(11): p. 1249-56.

16. Jung, D.W., et al., *Tumor-stromal crosstalk in invasion of oral squamous cell carcinoma: a pivotal role of CCL7.* Int J Cancer, 2010. **127**(2): p. 332-44.

17. Lee, Y.S., et al., *Crosstalk between CCL7 and CCR3 promotes metastasis of colon cancer cells via ERK-JNK signaling pathways.* Oncotarget, 2016. **7**(24): p. 36842-36853.

18. Dempe, S., et al., *Antitumoral activity of parvovirus-mediated IL-2 and MCP-3/CCL7 delivery into human pancreatic cancer: implication of leucocyte recruitment.* Cancer Immunol Immunother, 2012. **61**(11): p. 2113-23.

19. Ehrig, K., et al., *Growth inhibition of different human colorectal cancer xenografts after a single intravenous injection of oncolytic vaccinia virus GLV-1h68.* J Transl Med, 2013. **11**: p. 79.

20. Bian, G., et al., *Interferon-Gamma Receptor Signaling Plays an Important Role in Restraining Murine Ovarian Tumor Progression.* J Immunol Res Ther, 2016. **1**(1): p. 15-21.

21. Farmaki, E., et al., *A CCL8 gradient drives breast cancer cell dissemination.* Oncogene, 2016. **35**(49): p. 6309-6318.

22. Halvorsen, E.C., et al., *Maraviroc decreases CCL8-mediated migration of CCR5(+) regulatory T cells and reduces metastatic tumor growth in the lungs.* Oncoimmunology, 2016. **5**(6): p. e1150398.

23. Barbai, T., et al., *The importance of microenvironment: the role of CCL8 in metastasis formation of melanoma.* Oncotarget, 2015. **6**(30): p. 29111-28.

24. Jaguin, M., et al., *Polarization profiles of human M-CSF-generated macrophages and comparison of M1-markers in classically activated macrophages from GM-CSF and M-CSF origin.* Cell Immunol, 2013. **281**(1): p. 51-61.

25. Mantovani, A., et al., *Macrophage polarization: tumor-associated macrophages as a paradigm for polarized M2 mononuclear phagocytes.* Trends Immunol, 2002. **23**(11): p. 549-55.

26. Mizukami, Y., et al., *CCL17 and CCL22 chemokines within tumor microenvironment are related to accumulation of Foxp3+ regulatory T cells in gastric cancer.* Int J Cancer, 2008. **122**(10): p. 2286-93.

27. Lane, D., et al., *CCL18 from ascites promotes ovarian cancer cell migration through proline-rich tyrosine kinase 2 signaling.* Mol Cancer, 2016. **15**(1): p. 58.

28. Chen, J., et al., *CCL18 from tumor-associated macrophages promotes breast cancer metastasis via PITPNM3.* Cancer Cell, 2011. **19**(4): p. 541-55.

29. Noor, S., et al., *CCR7-dependent immunity during acute Toxoplasma gondii infection.* Infect Immun, 2010. **78**(5): p. 2257-63.

30. Zhang, Q., et al., *CCL19/CCR7 upregulates heparanase via specificity protein-1 (Sp1) to promote invasion of cell in lung cancer.* Tumour Biol, 2013. **34**(5): p. 2703-8.

31. Zhang, X., et al., *Increased CCL19 expression is associated with progression in cervical cancer.* Oncotarget, 2017. **8**(43): p. 73817-73825.

32. Hillinger, S., et al., *CCL19 reduces tumour burden in a model of advanced lung cancer.* Br J Cancer, 2006. **94**(7): p. 1029-34.

33. Hwang, H., et al., *Human breast cancer-derived soluble factors facilitate CCL19-induced chemotaxis of human dendritic cells.* Sci Rep, 2016. **6**: p. 30207.

34. Xiong, Y., et al., *CCL21/CCR7 interaction promotes cellular migration and invasion via modulation of the MEK/ERK1/2 signaling pathway and correlates with lymphatic metastatic spread and poor prognosis in urinary bladder cancer.* Int J Oncol, 2017. **51**(1): p. 75-90.

35. Tutunea-Fatan, E., et al., *The role of CCL21/CCR7 chemokine axis in breast cancer-induced lymphangiogenesis.* Mol Cancer, 2015. **14**: p. 35.

36. Curiel, T.J., et al., *Specific recruitment of regulatory T cells in ovarian carcinoma fosters immune privilege and predicts reduced survival.* Nature Medicine, 2004. **10**(9): p. 942-949.

37. Wiedemann, G.M., et al., *Cancer cell-derived IL-1alpha induces CCL22 and the recruitment of regulatory T cells.* Oncoimmunology, 2016. **5**(9): p. e1175794.

38. Jin, L., et al., *CCL24 contributes to HCC malignancy via RhoB- VEGFA-VEGFR2 angiogenesis pathway and indicates poor prognosis.* Oncotarget, 2017. **8**(3): p. 5135-5148.

39. Asegaonkar, S.B., et al., *C-Reactive Protein and Breast Cancer: New Insights from Old Molecule.* Int J Breast Cancer, 2015. **2015**: p. 145647.

40. Allin, K.H. and B.G. Nordestgaard, *Elevated C-reactive protein in the diagnosis, prognosis, and cause of cancer.* Crit Rev Clin Lab Sci, 2011. **48**(4): p. 155-70.

41. Yang, J., et al., *Human C-reactive protein binds activating Fcgamma receptors and protects myeloma tumor cells from apoptosis.* Cancer Cell, 2007. **12**(3): p. 252-65.

42. Sallusto, F., A. Lanzavecchia, and C.R. Mackay, *Chemokines and chemokine receptors in T-cell priming and Th1/Th2-mediated responses.* Immunol Today, 1998. **19**(12): p. 568-74.

43. Bronger, H., et al., *CXCL9 and CXCL10 predict survival and are regulated by cyclooxygenase inhibition in advanced serous ovarian cancer.* Br J Cancer, 2016. **115**(5): p. 553-63.

44. Guirnalda, P., et al., *Interferon gamma-induced intratumoral expression of CXCL9 alters the local distribution of T cells following immunotherapy with Listeria monocytogenes.* Oncoimmunology, 2013. **2**(8): p. e25752.

45. Gorbachev, A.V., et al., *CXC chemokine ligand 9/monokine induced by IFN-gamma production by tumor cells is critical for T cell-mediated suppression of cutaneous tumors.* J Immunol, 2007. **178**(4): p. 2278-86.

46. Liu, Z., et al., *CXCL11-Armed oncolytic poxvirus elicits potent antitumor immunity and shows enhanced therapeutic efficacy.* Oncoimmunology, 2016. **5**(3): p. e1091554.

47. Namkoong, H., et al., *Enhancement of antigen-specific CD8 T cell responses by co-delivery of Fc-fused CXCL11.* Vaccine, 2014. **32**(10): p. 1205-12.

48. Hensbergen, P.J., et al., *The CXCR3 targeting chemokine CXCL11 has potent antitumor activity in vivo involving attraction of CD8+ T lymphocytes but not inhibition of angiogenesis.* J Immunother, 2005. **28**(4): p. 343-51.

49. Kulbe, H., et al., *A dynamic inflammatory cytokine network in the human ovarian cancer microenvironment.* Cancer Res, 2012. **72**(1): p. 66-75.

50. Givel, A.M., et al., *miR200-regulated CXCL12beta promotes fibroblast heterogeneity and immunosuppression in ovarian cancers.* Nat Commun, 2018. **9**(1): p. 1056.

51. Righi, E., et al., *CXCL12/CXCR4 blockade induces multimodal antitumor effects that prolong survival in an immunocompetent mouse model of ovarian cancer.* Cancer Res, 2011. **71**(16): p. 5522-5534.

52. Takagi, R., et al., *B cell chemoattractant CXCL13 is preferentially expressed by human Th17 cell clones.* J Immunol, 2008. **181**(1): p. 186-9.

53. Singh, R., et al., *Expression and clinical significance of CXCR5/CXCL13 in human nonsmall cell lung carcinoma.* Int J Oncol, 2014. **45**(6): p. 2232-40.

54. Biswas, S., et al., *CXCL13-CXCR5 co-expression regulates epithelial to mesenchymal transition of breast cancer cells during lymph node metastasis.* Breast Cancer Res Treat, 2014. **143**(2): p. 265-76.

55. Razis, E., et al., *Improved outcome of high-risk early HER2 positive breast cancer with high CXCL13-CXCR5 messenger RNA expression.* Clin Breast Cancer, 2012. **12**(3): p. 183-93.

56. Yamada, H., et al., *Th1 is the predominant helper T cell subset that produces GM-CSF in the joint of rheumatoid arthritis.* RMD Open, 2017. **3**(1): p. e000487.

57. Grifka-Walk, H.M., D.A. Giles, and B.M. Segal, *IL-12-polarized Th1 cells produce GM-CSF and induce EAE independent of IL-23.* Eur J Immunol, 2015. **45**(10): p. 2780-6.

58. Reggiani, F., et al., *Adipose Progenitor Cell Secretion of GM-CSF and MMP9 Promotes a Stromal and Immunological Microenvironment That Supports Breast Cancer Progression.* Cancer Res, 2017. **77**(18): p. 5169-5182.

59. Gutschalk, C.M., et al., *Granulocyte colony-stimulating factor and granulocyte-macrophage colony-stimulating factor promote malignant growth of cells from head and neck squamous cell carcinomas in vivo.* Cancer Res, 2006. **66**(16): p. 8026-36.

60. Nebiker, C.A., et al., *GM-CSF Production by Tumor Cells Is Associated with Improved Survival in Colorectal Cancer.* Clin Cancer Res, 2014. **20**(12): p. 3094-106.

61. Masoud, G.N. and W. Li, *HIF-1α pathway: role, regulation and intervention for cancer therapy.* Acta Pharmaceutica Sinica B, 2015. **5**(5): p. 378-389.

62. Palazon, A., et al., *HIF transcription factors, inflammation, and immunity.* Immunity, 2014. **41**(4): p. 518-28.

63. Huang, et al., *HIF-1-mediated suppression of acyl-CoA dehydrogenases and fatty acid oxidation is critical for cancer progression.* Cell Rep, 2014. **8**(6): p. 1930-1942.

64. Bensaad, K., et al., *Fatty acid uptake and lipid storage induced by HIF-1alpha contribute to cell growth and survival after hypoxia-reoxygenation.* Cell Rep, 2014. **9**(1): p. 349-365.

65. McGhee, J.R., *The world of TH1/TH2 subsets: first proof.* J Immunol, 2005. **175**(1): p. 3-4.

66. Romagnani, S., *Th1/Th2 cells.* Inflamm Bowel Dis, 1999. **5**(4): p. 285-94.

67. Hayakawa, Y., et al., *IFN-gamma-mediated inhibition of tumor angiogenesis by natural killer T-cell ligand, alpha-galactosylceramide.* Blood, 2002. **100**(5): p. 1728-33.

68. Petretto, A., et al., *Proteomic analysis uncovers common effects of IFN-gamma and IL-27 on the HLA class I antigen presentation machinery in human cancer cells.* Oncotarget, 2016. **7**(45): p. 72518-72536.

69. Gabay, C., C. Lamacchia, and G. Palmer, *IL-1 pathways in inflammation and human diseases.* Nat Rev Rheumatol, 2010. **6**(4): p. 232-41.

70. Voronov, E., et al., *Unique Versus Redundant Functions of IL-1alpha and IL-1beta in the Tumor Microenvironment.* Front Immunol, 2013. **4**: p. 177.

71. Carmi, Y., et al., *The role of IL-1beta in the early tumor cell-induced angiogenic response.* J Immunol, 2013. **190**(7): p. 3500-9.

72. Schauer, I.G., et al., *Interleukin-1beta promotes ovarian tumorigenesis through a p53/NF-kappaB-mediated inflammatory response in stromal fibroblasts.* Neoplasia, 2013. **15**(4): p. 409-20.

73. Voigt, C., et al., *Cancer cells induce interleukin-22 production from memory CD4(+) T cells via interleukin-1 to promote tumor growth.* Proc Natl Acad Sci U S A, 2017. **114**(49): p. 12994-12999.

74. Wang, K.S., D.A. Frank, and J. Ritz, *Interleukin-2 enhances the response of natural killer cells to interleukin-12 through up-regulation of the interleukin-12 receptor and STAT4.* Blood, 2000. **95**(10): p. 3183-90.

75. Gasteiger, G., et al., *IL-2-dependent tuning of NK cell sensitivity for target cells is controlled by regulatory T cells.* J Exp Med, 2013. **210**(6): p. 1167-78.

76. Tomala, J., et al., *In vivo expansion of activated naive CD8+ T cells and NK cells driven by complexes of IL-2 and anti-IL-2 monoclonal antibody as novel approach of cancer immunotherapy.* J Immunol, 2009. **183**(8): p. 4904-12.

77. Yanagida, M., et al., *Effects of T-helper 2-type cytokines, interleukin-3 (IL-3), IL-4, IL-5, and IL-6 on the survival of cultured human mast cells.* Blood, 1995. **86**(10): p. 3705-14.

78. Dentelli, P., et al., *IL-3 is a novel target to interfere with tumor vasculature.* Oncogene, 2011. **30**(50): p. 4930-40.

79. Pyarajan, S., et al., *Interleukin-3 (IL-3)-induced c-fos activation is modulated by Gab2-calcineurin interaction.* J Biol Chem, 2008. **283**(35): p. 23505-9.

80. Parada, Y., et al., *BCR-ABL and interleukin 3 promote haematopoietic cell proliferation and survival through modulation of cyclin D2 and p27Kip1 expression.* J Biol Chem, 2001. **276**(26): p. 23572-80.

81. Lee, J.W., et al., *IL-3 expression by myeloma cells increases both osteoclast formation and growth of myeloma cells.* Blood, 2004. **103**(6): p. 2308-15.

82. Lombardo, G., et al., *IL-3R-alpha blockade inhibits tumor endothelial cell-derived extracellular vesicle (EV)-mediated vessel formation by targeting the beta-catenin pathway.* Oncogene, 2018. **37**(9): p. 1175-1191.

83. Martinez, F.O. and S. Gordon, *The M1 and M2 paradigm of macrophage activation: time for reassessment.* F1000Prime Rep, 2014. **6**: p. 13.

84. Martinez, F.O., et al., *Macrophage activation and polarization.* Front Biosci, 2008. **13**: p. 453-61.

85. Mantovani, A., et al., *The chemokine system in diverse forms of macrophage activation and polarization.* Trends Immunol, 2004. **25**(12): p. 677-86.

86. Gooch, J.L., A.V. Lee, and D. Yee, *Interleukin 4 inhibits growth and induces apoptosis in human breast cancer cells.* Cancer Res, 1998. **58**(18): p. 4199-205.

87. Lee, I.Y., et al., *Interleukin-4 inhibits the vascular endothelial growth factor- and basic fibroblast growth factor-induced angiogenesis in vitro.* Mol Cells, 2002. **14**(1): p. 115-21.

88. Eguchi, J., et al., *IL-4-transfected tumor cell vaccines activate tumor-infiltrating dendritic cells and promote type-1 immunity.* J Immunol, 2005. **174**(11): p. 7194-201.

89. Stoppacciaro, A., et al., *Genetic modification of a carcinoma with the IL-4 gene increases the influx of dendritic cells relative to other cytokines.* Eur J Immunol, 1997. **27**(9): p. 2375-82.

90. Tepper, R.I., P.K. Pattengale, and P. Leder, *Murine interleukin-4 displays potent anti-tumor activity in vivo.* Cell, 1989. **57**(3): p. 503-12.

91. Lee, E.J., et al., *Interleukin-5 enhances the migration and invasion of bladder cancer cells via ERK1/2-mediated MMP-9/NF-kappaB/AP-1 pathway: involvement of the p21WAF1 expression.* Cell Signal, 2013. **25**(10): p. 2025-38.

92. Zaynagetdinov, R., et al., *Interleukin-5 facilitates lung metastasis by modulating the immune microenvironment.* Cancer Res, 2015. **75**(8): p. 1624-1634.

93. Kumari, N., et al., *Role of interleukin-6 in cancer progression and therapeutic resistance.* Tumour Biol, 2016. **37**(9): p. 11553-11572.

94. Scheller, J., et al., *The pro- and anti-inflammatory properties of the cytokine interleukin-6.* Biochim Biophys Acta, 2011. **1813**(5): p. 878-88.

95. Kortylewski, M., et al., *Inhibiting Stat3 signaling in the hematopoietic system elicits multicomponent antitumor immunity.* Nat Med, 2005. **11**(12): p. 1314-21.

96. Herrmann, A., et al., *Targeting Stat3 in the myeloid compartment drastically improves the in vivo antitumor functions of adoptively transferred T cells.* Cancer Res, 2010. **70**(19): p. 7455-64.

97. Gotthardt, D., et al., *Loss of STAT3 in murine NK cells enhances NK cell-dependent tumor surveillance.* Blood, 2014. **124**(15): p. 2370-9.

98. Yu, H., D. Pardoll, and R. Jove, *STATs in cancer inflammation and immunity: a leading role for STAT3.* Nat Rev Cancer, 2009. **9**(11): p. 798-809.

99. Waugh, D.J. and C. Wilson, *The interleukin-8 pathway in cancer.* Clin Cancer Res, 2008. **14**(21): p. 6735-41.

100. Inoue, K., et al., *Interleukin 8 expression regulates tumorigenicity and metastases in androgen-independent prostate cancer.* Clin Cancer Res, 2000. **6**(5): p. 2104-19.

101. Mian, B.M., et al., *Fully human anti-interleukin 8 antibody inhibits tumor growth in orthotopic bladder cancer xenografts via down-regulation of matrix metalloproteases and nuclear factor-kappaB.* Clin Cancer Res, 2003. **9**(8): p. 3167-75.

102. Karashima, T., et al., *Nuclear factor-kappaB mediates angiogenesis and metastasis of human bladder cancer through the regulation of interleukin-8.* Clin Cancer Res, 2003. **9**(7): p. 2786-97.

103. Huang, S., et al., *Fully humanized neutralizing antibodies to interleukin-8 (ABX-IL8) inhibit angiogenesis, tumor growth, and metastasis of human melanoma.* Am J Pathol, 2002. **161**(1): p. 125-34.

104. Lu, Y., et al., *Th9 cells promote antitumor immune responses in vivo.* J Clin Invest, 2012. **122**(11): p. 4160-71.

105. You, F.P., et al., *Th9 cells promote antitumor immunity via IL-9 and IL-21 and demonstrate atypical cytokine expression in breast cancer.* Int Immunopharmacol, 2017. **52**: p. 163-167.

106. Appleby, L.J., et al., *Sources of heterogeneity in human monocyte subsets.* Immunol Lett, 2013. **152**(1): p. 32-41.

107. Qi, L., et al., *IL-10 secreted by M2 macrophage promoted tumorigenesis through interaction with JAK2 in glioma.* Oncotarget, 2016. **7**(44): p. 71673-71685.

108. Emmerich, J., et al., *IL-10 directly activates and expands tumor-resident CD8(+) T cells without de novo infiltration from secondary lymphoid organs.* Cancer Res, 2012. **72**(14): p. 3570-81.

109. Emmerich, J., J.B. Mumm, and M. Oft, *Autochthonous T cells to the rescue: IL-10 directly activates tumor-resident CD8(+) T cells.* Oncoimmunology, 2012. **1**(9): p. 1637-1639.

110. Mumm, J.B., et al., *IL-10 elicits IFNgamma-dependent tumor immune surveillance.* Cancer Cell, 2011. **20**(6): p. 781-96.

111. Zeh, H.J., 3rd, et al., *Interleukin-12 promotes the proliferation and cytolytic maturation of immune effectors: implications for the immunotherapy of cancer.* J Immunother Emphasis Tumor Immunol, 1993. **14**(2): p. 155-61.

112. Teng, M.W., et al., *IL-12 and IL-23 cytokines: from discovery to targeted therapies for immune-mediated inflammatory diseases.* Nat Med, 2015. **21**(7): p. 719-29.

113. Kerkar, S.P., et al., *IL-12 triggers a programmatic change in dysfunctional myeloid-derived cells within mouse tumors.* J Clin Invest, 2011. **121**(12): p. 4746-57.

114. Voest, E.E., et al., *Inhibition of angiogenesis in vivo by interleukin 12.* J Natl Cancer Inst, 1995. **87**(8): p. 581-6.

115. Fujisawa, T., et al., *A novel role of interleukin-13 receptor alpha2 in pancreatic cancer invasion and metastasis.* Cancer Res, 2009. **69**(22): p. 8678-85.

116. Fujisawa, T., B.H. Joshi, and R.K. Puri, *IL-13 regulates cancer invasion and metastasis through IL-13Ralpha2 via ERK/AP-1 pathway in mouse model of human ovarian cancer.* Int J Cancer, 2012. **131**(2): p. 344-56.

117. Barderas, R., et al., *High expression of IL-13 receptor alpha2 in colorectal cancer is associated with invasion, liver metastasis, and poor prognosis.* Cancer Res, 2012. **72**(11): p. 2780-90.

118. Punt, S., et al., *The correlations between IL-17 vs. Th17 cells and cancer patient survival: a systematic review.* Oncoimmunology, 2015. **4**(2): p. e984547.

119. Sutton, C.E., et al., *Interleukin-1 and IL-23 induce innate IL-17 production from gammadelta T cells, amplifying Th17 responses and autoimmunity.* Immunity, 2009. **31**(2): p. 331-41.

120. Gaffen, S.L., et al., *The IL-23-IL-17 immune axis: from mechanisms to therapeutic testing.* Nat Rev Immunol, 2014. **14**(9): p. 585-600.

121. Cai, Y., et al., *Pivotal role of dermal IL-17-producing gammadelta T cells in skin inflammation.* Immunity, 2011. **35**(4): p. 596-610.

122. Rei, M., et al., *Murine CD27(-) Vgamma6(+) gammadelta T cells producing IL-17A promote ovarian cancer growth via mobilization of protumor small peritoneal macrophages.* Proc Natl Acad Sci U S A, 2014. **111**(34): p. E3562-70.

123. Markowitz, G.J., et al., *Inflammation-Dependent IL18 Signaling Restricts Hepatocellular Carcinoma Growth by Enhancing the Accumulation and Activity of Tumor-Infiltrating Lymphocytes.* Cancer Res, 2016. **76**(8): p. 2394-405.

124. Li, S., et al., *TLR2 limits development of hepatocellular carcinoma by reducing IL18-mediated immunosuppression.* Cancer Res, 2015. **75**(6): p. 986-95.

125. Ma, Z., et al., *Augmentation of Immune Checkpoint Cancer Immunotherapy with IL18.* Clin Cancer Res, 2016. **22**(12): p. 2969-80.

126. Wei, L., et al., *IL-21 is produced by Th17 cells and drives IL-17 production in a STAT3-dependent manner.* J Biol Chem, 2007. **282**(48): p. 34605-10.

127. Peluso, I., et al., *IL-21 counteracts the regulatory T cell-mediated suppression of human CD4+ T lymphocytes.* J Immunol, 2007. **178**(2): p. 732-9.

128. Kannappan, V., et al., *Interleukin 21 inhibits cancer-mediated FOXP3 induction in naive human CD4 T cells.* Cancer Immunol Immunother, 2017. **66**(5): p. 637-645.

129. Attridge, K., et al., *IL-21 promotes CD4 T cell responses by phosphatidylinositol 3-kinase-dependent upregulation of CD86 on B cells.* J Immunol, 2014. **192**(5): p. 2195-201.

130. Liang, S.C., et al., *Interleukin (IL)-22 and IL-17 are coexpressed by Th17 cells and cooperatively enhance expression of antimicrobial peptides.* J Exp Med, 2006. **203**(10): p. 2271-9.

131. Fukui, H., et al., *IL-22 produced by cancer-associated fibroblasts promotes gastric cancer cell invasion via STAT3 and ERK signaling.* Br J Cancer, 2014. **111**(4): p. 763-71.

132. Verreck, F.A., et al., *Human IL-23-producing type 1 macrophages promote but IL-10-producing type 2 macrophages subvert immunity to (myco)bacteria.* Proc Natl Acad Sci U S A, 2004. **101**(13): p. 4560-5.

133. Mantovani, A., A. Sica, and M. Locati, *Macrophage polarization comes of age.* Immunity, 2005. **23**(4): p. 344-6.

134. Helbling, M., et al., *Investigation of IL-23 (p19, p40) and IL-23R identifies nuclear expression of IL-23 p19 as a favorable prognostic factor in colorectal cancer: a retrospective multicenter study of 675 patients.* Oncotarget, 2014. **5**(13): p. 4671-82.

135. Grivennikov, S.I., et al., *Adenoma-linked barrier defects and microbial products drive IL-23/IL-17-mediated tumour growth.* Nature, 2012. **491**(7423): p. 254-8.

136. Langowski, J.L., et al., *IL-23 promotes tumour incidence and growth.* Nature, 2006. **442**(7101): p. 461-465.

137. Jovanovic, I.P., et al., *Interleukin-33/ST2 axis promotes breast cancer growth and metastases by facilitating intratumoral accumulation of immunosuppressive and innate lymphoid cells.* Int J Cancer, 2014. **134**(7): p. 1669-82.

138. Kim, J.Y., et al., *Interleukin-33/ST2 axis promotes epithelial cell transformation and breast tumorigenesis via upregulation of COT activity.* Oncogene, 2015. **34**(38): p. 4928-38.

139. Han, L., et al., *Interleukin-33 promotes inflammation-induced lymphangiogenesis via ST2/TRAF6-mediated Akt/eNOS/NO signalling pathway.* Sci Rep, 2017. **7**(1): p. 10602.

140. Fang, M., et al., *IL33 Promotes Colon Cancer Cell Stemness via JNK Activation and Macrophage Recruitment.* Cancer Res, 2017. **77**(10): p. 2735-2745.

141. Gresnigt, M.S. and F.L. van de Veerdonk, *Biology of IL-36 cytokines and their role in disease.* Semin Immunol, 2013. **25**(6): p. 458-65.

142. Wang, X., et al., *IL-36gamma Transforms the Tumor Microenvironment and Promotes Type 1 Lymphocyte-Mediated Antitumor Immune Responses.* Cancer Cell, 2015. **28**(3): p. 296-306.

143. Josephs, D.H., et al., *Anti-Folate Receptor-alpha IgE but not IgG Recruits Macrophages to Attack Tumors via TNFalpha/MCP-1 Signaling.* Cancer Res, 2017.

144. Gu, L., et al., *Control of TH2 polarization by the chemokine monocyte chemoattractant protein-1.* Nature, 2000. **404**(6776): p. 407-11.

145. Andrae, J., R. Gallini, and C. Betsholtz, *Role of platelet-derived growth factors in physiology and medicine.* Genes & Development, 2008. **22**(10): p. 1276-1312.

146. Ding, W., et al., *Platelet-derived growth factor (PDGF)-PDGF receptor interaction activates bone marrow-derived mesenchymal stromal cells derived from chronic lymphocytic leukemia: implications for an angiogenic switch.* Blood, 2010. **116**(16): p. 2984-93.

147. Sahraei, M., et al., *MUC1 regulates PDGFA expression during pancreatic cancer progression.* Oncogene, 2012. **31**(47): p. 4935-45.

148. Ludviksson, B.R., et al., *The effect of TGF-beta1 on immune responses of naive versus memory CD4+ Th1/Th2 T cells.* Eur J Immunol, 2000. **30**(7): p. 2101-11.

149. Maeda, H. and A. Shiraishi, *TGF-beta contributes to the shift toward Th2-type responses through direct and IL-10-mediated pathways in tumor-bearing mice.* J Immunol, 1996. **156**(1): p. 73-8.

150. Bellomo, C., L. Caja, and A. Moustakas, *Transforming growth factor beta as regulator of cancer stemness and metastasis.* Br J Cancer, 2016. **115**(7): p. 761-9.

151. Fan, Q.M., et al., *Tumor-associated macrophages promote cancer stem cell-like properties via transforming growth factor-beta1-induced epithelial-mesenchymal transition in hepatocellular carcinoma.* Cancer Lett, 2014. **352**(2): p. 160-8.

152. Bierie, B. and H.L. Moses, *Transforming growth factor beta (TGF-beta) and inflammation in cancer.* Cytokine Growth Factor Rev, 2010. **21**(1): p. 49-59.

153. Vinals, F. and J. Pouyssegur, *Transforming growth factor beta1 (TGF-beta1) promotes endothelial cell survival during in vitro angiogenesis via an autocrine mechanism implicating TGF-alpha signaling.* Mol Cell Biol, 2001. **21**(21): p. 7218-30.

154. Zhao, M., et al., *Interleukin 37 promotes angiogenesis through TGF-beta signaling.* Sci Rep, 2017. **7**(1): p. 6113.

155. Daroqui, M.C., et al., *TGF-beta autocrine pathway and MAPK signaling promote cell invasiveness and in vivo mammary adenocarcinoma tumor progression.* Oncol Rep, 2012. **28**(2): p. 567-75.

156. Tosh, K.W., et al., *The IL-12 Response of Primary Human Dendritic Cells and Monocytes to Toxoplasma gondii Is Stimulated by Phagocytosis of Live Parasites Rather Than Host Cell Invasion.* J Immunol, 2016. **196**(1): p. 345-56.

157. Quan, J.H., et al., *Induction of protective immune responses by a multiantigenic DNA vaccine encoding GRA7 and ROP1 of Toxoplasma gondii.* Clin Vaccine Immunol, 2012. **19**(5): p. 666-74.

158. Vouldoukis, I., et al., *IgE mediates killing of intracellular Toxoplasma gondii by human macrophages through CD23-dependent, interleukin-10 sensitive pathway.* PLoS One, 2011. **6**(4): p. e18289.

159. Josephs, D.H., et al., *Anti-Folate Receptor-alpha IgE but not IgG Recruits Macrophages to Attack Tumors via TNFalpha/MCP-1 Signaling.* Cancer Res, 2017. **77**(5): p. 1127-1141.

160. Baxevanis, C.N., et al., *Compromised anti-tumor responses in tumor necrosis factor-alpha knockout mice.* Eur J Immunol, 2000. **30**(7): p. 1957-66.

161. Wang, R., et al., *Natural killer cell-produced IFN-gamma and TNF-alpha induce target cell cytolysis through up-regulation of ICAM-1.* J Leukoc Biol, 2012. **91**(2): p. 299-309.

162. Daller, B., et al., *Lymphotoxin-beta receptor activation by lymphotoxin-alpha(1)beta(2) and LIGHT promotes tumor growth in an NFkappaB-dependent manner.* Int J Cancer, 2011. **128**(6): p. 1363-70.

163. Zhou, P., et al., *Targeting lymphotoxin-mediated negative selection to prevent prostate cancer in mice with genetic predisposition.* Proc Natl Acad Sci U S A, 2009. **106**(40): p. 17134-9.

164. Hehlgans, T., et al., *Lymphotoxin-beta receptor immune interaction promotes tumor growth by inducing angiogenesis.* Cancer Res, 2002. **62**(14): p. 4034-40.

165. Carmeliet, P., *VEGF as a Key Mediator of Angiogenesis in Cancer.* Oncology, 2005. **69**(3): p. 4-10.

166. Goel, H.L. and A.M. Mercurio, *VEGF targets the tumour cell.* Nat Rev Cancer, 2013. **13**(12): p. 871-82.

167. Terme, M., et al., *VEGFA-VEGFR pathway blockade inhibits tumor-induced regulatory T-cell proliferation in colorectal cancer.* Cancer Res, 2013. **73**(2): p. 539-49.

168. Cao, Y., et al., *VEGF exerts an angiogenesis-independent function in cancer cells to promote their malignant progression.* Cancer Res, 2012. **72**(16): p. 3912-8.
