# Supplementary material for: Immune mediator expression signatures are associated with improved outcome in ovarian carcinoma
Source: Oncoimmunology. 2019 Mar 28;8(6):e1593811. doi: 10.1080/2162402X.2019.1593811 (PMC6492968; doi:10.1080/2162402X.2019.1593811)
Supplement: Supplemental Material [file koni-08-06-1593811-s001.zip › Supplementary Table 2(1).docx]

**Supplementary Table 2. Metrics derived from confusion matrix of Random Forest, Support Vector Machine, and Neural Network using “All genes” or “44 genes”.** The average of 5 runs is shown here, where the range is from 0 to 1 (1 = 100%).

|  | **Matthew Correlation Coefficient** | **F1_score** | **Precision** | **Recall** | **Specificity** |
| --- | --- | --- | --- | --- | --- |
|  | All genes | | | | |
| Random Forest | 0.9822058 | 0.9975904 | 0.9975904 | 0.9975904 | 0.9846154 |
| Support Vector Machine | 0.9072424 | 0.9893854 | 0.983685 | 0.9951808 | 0.8717084 |
| Neural Network | 0.918246 | 0.98923 | 0.9975308 | 0.9812214 | 0.9846154 |
|  | 44 genes | | | | |
| Random Forest | 0.8870536 | 0.98269524 | 0.97958056 | 0.98603636 | 0.86282532 |
| Support Vector Machine | 0.8519182 | 0.9794654 | 0.9904118 | 0.969175 | 0.9292062 |
| Neural Network | 0.8581766 | 0.9792192 | 0.9926058 | 0.9664768 | 0.9490476 |
